# Deregulation of the imprinted DLK1-DIO3 locus ncRNAs is associated with replicative senescence of human adipose-derived stem cells

**DOI:** 10.1371/journal.pone.0206534

**Published:** 2018-11-05

**Authors:** Silvia García-López, Carmen Albo-Castellanos, Rocio G. Urdinguio, Susana Cañón, Fátima Sánchez-Cabo, Alberto Martínez-Serrano, Mario F. Fraga, Antonio Bernad

**Affiliations:** 1 Department of Immunology and Oncology, Centro Nacional de Biotecnología (CNB-CSIC), Campus de Cantoblanco, Madrid, Spain; 2 Department of Cardiovascular Development and Repair, Centro Nacional de Investigaciones Carlos III (CNIC), Madrid, Spain; 3 Cancer Epigenetics Laboratory, Institute of Oncology of Asturias (IUOPA), Hospital Universitaria Central de Asturias (HUCA) and Nanomaterials and Nanotechnology Research Center (CINN-CSIC), Universidad de Oviedo (UO), Asturias, Spain; 4 Bioinformatics Unit, Centro Nacional de Investigaciones Cardiovasculares Carlos III (CNIC), Madrid, Spain; 5 Molecular Biology Department (UAM) and Molecular Neuropathology Department, Center of Molecular Biology Severo Ochoa-CSIC, Universidad Autónoma de Madrid, Campus Cantoblanco, Madrid, Spain; Universitat des Saarlandes, GERMANY

## Abstract

**Background:**

Human adult adipose-derived stem cells (hADSCs) have become the most promising cell source for regenerative medicine. However the prolonged *ex vivo* expansion periods required to obtain the necessary therapeutic dose promotes progressive senescence, with the concomitant reduction of their therapeutic potential.

**Aim and scope:**

A better understanding of the determinants of hADSC senescence is needed to improve biosafety while preserving therapeutic efficiency. Here, we investigated the association between deregulation of the imprinted *DLK1-DIO3* region and replicative senescence in hADSC cultures.

**Methods:**

We compared hADSC cultures at short (P_S_) and prolonged (P_L_) passages, both in standard and low [O_2_] (21 and 3%, respectively), in relation to replicative senescence. hADSCs were evaluated for expression alterations in the *DLK1-DIO3* region on chromosome 14q32, and particularly in its main miRNA cluster.

**Results:**

Comparison of hADSCs cultured at P_L_ or P_S_ surprisingly showed a quite significant fraction (69%) of upregulated miRNAs in P_L_ cultures mapping to the imprinted 14q32 locus, the largest miRNA cluster described in the genome. In agreement, expression of the lncRNA *MEG3* (Maternally Expressed 3; *Meg3/Gtl*2), cultured at 21 and 3% [O_2_], was also significantly higher in P_L_ than in P_S_ passages. During hADSC replicative senescence the AcK16H4 activating mark was found to be significantly associated with the deregulation of the entire *DLK1-DIO3* locus, with a secondary regulatory role for the methylation of DMR regions.

**Conclusion:**

A direct relationship between *DLK1-DIO3* deregulation and replicative senescence of hADSCs is reported, involving upregulation of a very significant fraction of its largest miRNA cluster (14q32.31), paralleled by the progressive overexpression of the lncRNA *MEG3*, which plays a central role in the regulation of *Dlk1/Dio3* activation status in mice.

## Introduction

Human adipose-derived stem cells (hADSCs) have become an increasingly important cell source in regenerative medicine, as moderate yields can be obtained by minimally invasive techniques from different adipose depots. As the amount of cells obtained by this technique is, nevertheless, limited, *ex vivo* expansion is necessary for downstream clinical use. There is increasing evidence that standard cell culture methods at high [O_2_] (atmospheric; 21%) are stressful for several cell lineages, including hADSCs [1–4]. Indeed, in standard conditions, human mesenchymal stem cell (hMSC) cultures progressively evolve towards replicative senescence, with an accelerated rate of telomere erosion and an accumulation of genomic alterations [3–5]. Moreover, hADSC senescence correlates with the progressive loss of stem cell properties that could curtail their therapeutic potential [6–8]. Culture of hMSCs at lower concentrations (3–5%) of [O_2_] revealed a significant increase in culture lifespan and better cellular quality, which was associated with a favorable metabolic state of increased glycolysis and decreased oxidative phosphorylation, resulting in reduced levels of oxidative stress [3–8]. In agreement with these observations are the results of several studies using *ex vivo* treatments with antioxidants (reviewed in [9]); for example, short-term treatment of hADSCs with physiological concentrations of zinc sulphate promotes telomere length extension. It is important to note while low [O_2_] is usually referred to as hypoxia, low O_2_ tensions are considered normoxic in several organs [10,11].

Replicative senescence and aging involves a series of epigenetic alterations such as hypermethylation of specific genomic regions, but against a background of general DNA hypomethylation [12]. This scenario promotes silencing of proliferation-associated genes, whereas tumor suppressor genes are derepressed [13,14]. The main pathways activated in senescent cells are Rb/p16 and p53/21, leading to progressive cell cycle blockade and cell growth arrest. In line with this, the positive effects on hADSCs by short-term treatment with zinc sulphate are mediated by modulation of the methylation status of the promoter of TERT, the telomerase catalytic subunit [9]. Many studies consider senescence as a tumor suppressor network mechanism (reviewed in [15]).

The imprinted *DLK1-DIO3* domain at 14q32.2–32.31 has been associated with regulation of senescence and stem cell function, and is also involved in human cancer [16]. The *DLK1-DIO3* locus is conserved in placental mammals (*Dlk1/Dio3* in mice; located on 12qF1) and is one of the three imprinted loci (with IGF2/H19 and the 15q25.1 locus) that acquire their imprinting through the paternal allele [17,18]. In addition, *DLK1-DIO3* includes the largest cluster of miRNAs described thus far ([19]; see S1 Fig). Concerning senescence, several *DLK1-DIO3* mapping genes, both coding and non-coding, have been previously described to be able to regulate senescence in several cell types; for example, miR-369-5p and miR-485-5p have been shown to be involved in hADSC senescence [13,20,21]. Upregulation of *Dlk1/Dio3* miRNAs has also been implicated in murine lupus, being associated with global DNA hypomethylation, but with differential alterations in several splenic subsets [22]. Also, miR-679 and miR-300 are affected by long-lasting alterations in DNA methylation, as a result of fetal alcohol exposure [23]. In mouse cardiac progenitor cells, however, this correlation was the opposite: miR-300 is positively regulated by Bmi1, preventing senescence progression. Furthermore, miR-300 dowregulation is required for endothelial and cardiogenic differentiation [24].

*MEG3* (maternally expressed 3) is a maternally-expressed, imprinted long non-coding RNA (lncRNA) gene that maps to the *DLK1-DIO3* locus in humans, whereas the murine homologue (*Meg3/Gtl2*) maps to chromosome 12 [25]. *MEG3* has been extensively associated with multiple human cancer types and is proposed as a tumor suppressor and a negative regulator of angiogenesis [26,27]. Aberrant hypermethylation of the *MEG3* promoter is believed to be the main mechanism involved and its down-regulation is an unfavorable survival factor in bladder cancer [28,29]. *MEG3* has been also found in exosomes from cervicovaginal lavage of cancer patients [30]. Finally, It has been reported that *MEG3* is regulated by the retinoblastoma protein [31] and its forced expression usually suppresses proliferation and promotes apoptosis [32–34]. However, there is scant information on the role of ncRNAs mapping in the *DLK1-DIO3* locus in hADSC biology. In certain pathological conditions, such as the myelodysplastic syndromes, bone marrow hADSCs show an overactivation of the *DLK1-DIO3* locus and have prominent features of senescence, including a highly reduced osteogenic capacity [35]. In multiple myeloma, bone marrow hADSCs also exhibit increased senescence with participation of the overexpressed miR-485-5p, located in the *DLK1-DIO3* region [20].

Here, we have explored the possible association between adult hADSC replicative senescence and alterations in the regulation of the *DLK1-DIO3* locus.

## Materials and methods

### Human mesenchymal stem cell culture

hADSCs were cultured as described [3]; see Supporting Information.

### RT-qPCR analysis

Total RNA was isolated using TriReagent solution (Sigma). Complementary DNA (cDNA) was generated from 1 μg of total RNA using the SuperScript III First-Strand Synthesis System for RT-PCR kit (Invitrogen) in a 20-μl final reaction volume. Real-time PCR reactions were performed using 2 μl/well of a 1/5 dilution of each cDNA and 5 μl of Power SYBR Green PCR Master Mix (Applied Biosystems) in a 10 μl final volume. Results were analyzed using the comparative method (2^-ddCt^) and normalized to endogenous expression of ß-glucuronidase (GUSB).

### Microarray hybridization and data analysis

Total RNA extraction from eight biological samples (adult adipose tissue derived: short-term culture ahADSCs, *n* = 2 biological replicates; long-term culture ahADSCs, *n* = 2; pediatric adipose tissue-derived phADSCs, *n* = 3; long-term culture phADSCs, *n* = 1) was performed with the mirVana miRNA Isolation Kit (Applied Biosystems). Samples were hybridized to the human miRNA Microarray v1.0 (Agilent Technologies) and processed as indicated. miRNA data was normalized based on the VSN-invariant method. From a total of 721 human sequences, after normalization, only those probes with an average expression above the 20^th^ percentile of all average expressions (393 miRNAs) were considered for analysis. The *Limma* package was used to determine differentially expressed miRNAs, and we focused on those miRNAs with an adjusted p-value <0.2. miRNA data are accessible in the Gene Expression Omnibus (*GEO*) database repository (GSE121481).

### DNA methylation analysis

Genomic DNA was isolated using TriReagent solution and modified by sodium bisulfite conversion as described [36]. Briefly, 1 μg of genomic DNA was denaturated with a final concentration 0.3 M NaOH at 37°C for 10 minutes. Denaturated DNA was then treated with a final concentration of 2.6 M bisulphite/0.5 mM hydroquinone mixture, at 50°C for 16–18 hours, protected from light. Treated DNA was cleaned with the Wizard DNA clean-up system (Promega). Eluted samples were then treated with NaOH as above, and then ethanol-precipitated. PCR was performed on bisulfite-treated DNA, and PCR products were cloned into the pGEMT-Easy vector (Promega) and sequenced. To examine the methylation status of the IG-DMR and MEG-DMR, three and five clones, respectively, were analyzed for each condition. PCR primers are listed in Supplementary S4 Table and were previously reported [37]. Sequencing results were analyzed using CpGViewer software.

### Chromatin immunoprecipitation

Chromatin immunoprecipitation (ChIP) was performed as described [10]. Briefly, cultured cells were fixed with 1% formaldehyde and crosslinking was terminated by the addition of glycine to a final concentration of 125 mM. Cells were washed twice in cold PBS with protease inhibitors, scraped, collected, pelleted and resuspended in SDS lysis buffer (1% SDS, 10 mM EDTA, 50 mM Tris pH 8). Chromatin was fragmented using the Bioruptor Sonication System (Diagenode). Samples were immunoprecipitated using anti-IgG (ab46540, Abcam), -AcK16H4 (07–329, Millipore) or -total H3 antibodies (ab1791, Abcam). Samples were analyzed by quantitative PCR. Nonspecific adjustment (dCq) was calculated by (dCq = Cq_[IP]_ − Cq_[IgG]_). Fold enrichment was calculated as 2^(-ddCq), where ddCq is calculated by (ddCq = Cq_[PL]_ − Cq_[PS]_).

## Results

### *MEG3* expression is deregulated by replicative senescence in human ADSCs

**Fig 1:**
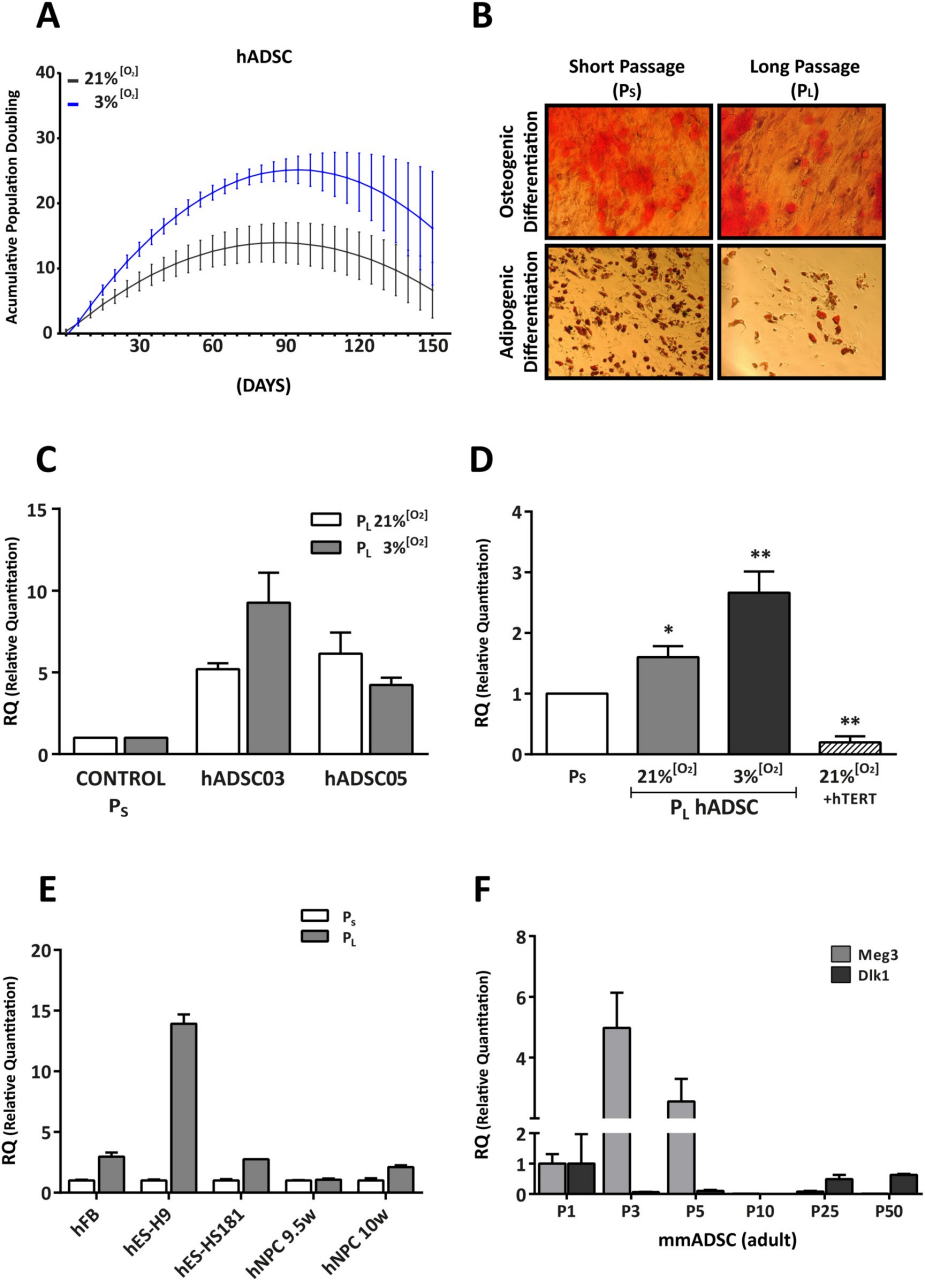
Replicative senescence is associated with upregulated lncRNA *MEG3* expression. (A) Model of hADSC proliferation kinetics. From the cumulative population doubling data for different hADSC cultures, we obtained a curve fitted to a polynomic function that modeled hADSC proliferation kinetics. The various hADSC cultures were used to establish a mean proliferation curve for 150 days. Each dot represents the mean ± SEM of data obtained by modeling the different hADSC cultures. (B) Representative images indicating loss of differentiation potential (adipogenic and osteogenic) in P_L_ cultures as compared with P_S_ cultures of hADSCs. (C) Relative quantitation of *MEG3* lncRNA in two different hADSC samples (*n* = 3 technical replicates) cultured for short (P_S_) and long (P_L_) periods. Data shown represent mean ± SEM. (D) Relative quantitation of *MEG3* expression in P_S_ and P_L_ hADSC cultures from a different source, Inbiobank (aADSC, *n* = 4 biological replicates; with overexpressed hTERT; +hTERT, *n* = 2 biological replicates); data represent mean ± SEM (* p <0.05, ** p <0.01, *** p <0.001; two-tailed paired t-test). (E) Relative quantitation of *MEG3* expression in different cell models (hFB, human fibroblasts; hES-H9, human embryonic stem cell H9; hES-HS181, human embryonic stem cell HS181; hNPC 9.5w, human neural precursor cells from a 9.5-week fetus; hNPC 10w, human neural precursor cells from a 10-week fetus); shown is a representative experiment with 3 technical replicates. (F) Relative quantitation of *Meg3* and *Dlk1* expression in mouse ADSCs (mADSCs) at various passages; shown is a representative experiment with 3 technical replicates. Data represent mean ± SEM.

We sought to compare the effects of standard (21% [O_2_]) *versus* normoxic (3% [O_2_]) conditions in prolonged (≥20 passages; but variable with the individual isolates) cultures of adult hADSCs (P_L_) and short (≤12 passages; P_S_) passages, in relation to the concurrent replicative senescence previously described [3–6]. We first confirmed that cell culture growth rate was higher at 3% [O_2_] than at 21% [O_2_] (Fig 1A, S1A and S1B Fig). Analysis of hADSCs grown at 21% [O_2_] or 3% [O_2_] showed that long passages led to a decreased capacity for differentiation (Fig 1B), to an increase in cell size and complexity (S1C Fig) and cellular morphology changes as well as SA-ß-gal positive staining (S1D Fig), as previously reported [3,4].

Because of the central role proposed for lncRNA *MEG3* in the regulation of the *Dlk1/Dio3* (S1E Fig) locus activation status in mouse [38], we investigated its potential deregulation with regards to replicative senescence in hADSCs. RT-qPCR analysis of *MEG3* expression in hADSCs cultured at 21 and 3% [O_2_] showed that *MEG3* expression was significantly higher in P_L_ than in P_S_ cultures (Fig 1C and 1D). To confirm the possible association of *MEG3* upregulation with replicative senescence, we analyzed the effect of constitutive expression (lentiviral transduction) of the telomerase catalytic subunit TERT, which hinders or prevents cell senescence in hADSCs [3,4]. The *MEG3* overexpression observed in P_L_ samples was significantly diminished in hADSCs expressing TERT, with levels below those found in P_S_ cultured hADSCs (Fig 1D). These data suggest that mechanisms involved in regulation of *MEG3* expression at the *DLK1-DIO3* locus are associated with replicative senescence. We also evaluated the behavior of another imprinted locus, *IGF2*, finding that its expression was also upregulated in P_L_ samples, and constitutive TERT expression prevented or counteracted this upregulation (S2A Fig). Overall, these findings suggest that multiple imprinted loci can be similarly affected during hADSC proliferative senescence.

To determine whether deregulation of the *DLK1-DIO3* locus could be a general feature of cell senescence, we analyzed *MEG3* expression in P_L_ and P_S_ cultures of several human cell lineages (Fig 1E). Similar to that found for hADSCs, *MEG3* was upregulated in human fibroblasts (hFBs) with cell passage. The human ES cell lines tested (H9, H181) showed large differences in *MEG3* expression after several passages, likely reflecting their heterogeneity, but both increased their levels of *MEG3* with progressive passaging. By contrast, neural progenitor cells (hNPCs) [39,40] showed discrete *MEG3* overexpression. Also, some v-myc-immortalized derivatives showed strong *MEG3* downregulation as compared with non-immortalized samples (S2B Fig). Because v-myc induces telomerase activity in hNPC lines [39,40] these results are compatible with our findings in hADSCs after hTERT overexpression (Fig 1D). In murine ADSCs, *Meg3* was upregulated in early passages (P2–P5) and then decreased significantly (Fig 1F), doubtless coupled to the culture growth crisis before immortalization. At later stages (P25–50), *Meg3* was re-expressed at a lower level relative to initial levels. Finally, expression of murine *Dlk1* (delta like non-canonical Notch ligand 1), also mapping in locus *Dlk1/Dio3*, did not parallel this profile (Fig 1F). In conclusion, our results suggest that upregulation of *MEG3* in association with replicative senescence seems to be a phenomenon mostly restricted to human primary adult cells, both hADSCs and hFBs.

### 14q32.31 miRNA cluster expression is globally altered by replicative senescence in human ADSCs

The significant alteration in *MEG3* expression prompted us to characterize the miRNA repertoire associated with replicative senescence in hADSCs. We focused mainly on the 14q32.31 miRNA cluster, the largest described in the genome (reviewed in [19]). hADSCs were cultured at 3% [O_2_], with the aim to reduce the artificial oxidative stress background generated by higher [O_2_]. The distribution of the normalized intensity of miRNA array data of hADSCs in P_L_ *versus* P_S_ cultures is depicted in S3A Fig. Statistical analysis identified 84 miRNAs (21.3%) differentially expressed in P_L_ *versus* P_S_ (S1 Table). Among the downregulated miRNAs [41], at least four members of the miR-17-92a cluster were identified (S1 Table). Interestingly, members of this cluster are oncomiRs, and their downregulation is associated with cell senescence and aging [14,41].

**Fig 2:**
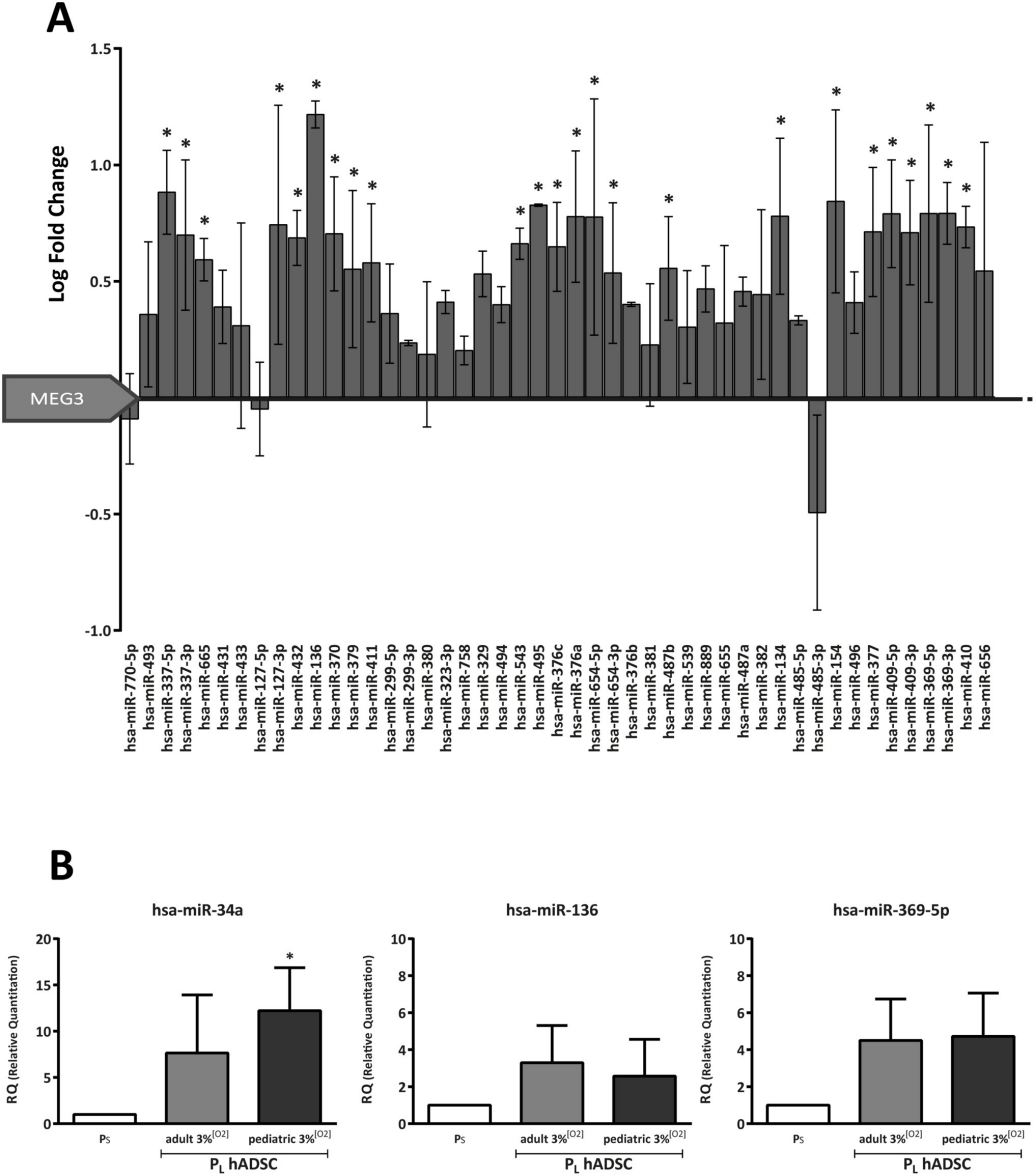
miRNA 14q32.31 cluster is deregulated in association with replicative senescence. (A) Bar graph shows log fold-change expression for the miRNAs in the 14q32 chromosome region that were deregulated in short-term (P_S_) *versus* long-term (P_L_) cultured adult ADSC samples (*n* = 2 biological replicates in both cases; mean ± SEM). (B) Relative quantitation (see extended methods in Supporting information for details) of selected miRNAs for array validation in adult (*n* = 3 biological replicates) and pediatric (*n* = 2 biological replicates) hADSCs; data represent mean ± SEM (* p <0.05; one-tailed paired t-test).

One significantly upregulated miRNA was miR-34a, which is considered a tumor suppressor and is reported to repress targets involved in cell cycle, apoptosis and senescence, following p53-induced transactivation [42,43]. Several other upregulated miRNAs found in our analysis have also been associated with cell senescence and aging [13,41]. Surprisingly, a quite significant fraction (69%) of upregulated miRNAs in hADSC P_L_ cultures (Fig 2A and S1 Table) mapped to the imprinted 14q32 locus (S1D Fig). Proportion statistical test analysis confirmed that the upregulated miRNA fraction in this region was significantly greater than that expected by chance (p <0.0001), therefore strongly suggesting a marked upregulation of the locus associated with replicative senescence. To clarify whether these changes could be associated with chronological aging, we performed a similar analysis in hADSCs obtained from pediatric (<15-year-old donors; phADSCs) that demonstrated a comparable *in vitro* behavior. After data normalization, all miRNAs of the 14q32 locus included in the miRNA array were found to be deregulated, with a predominant tendency to upregulation (S4A Fig).

We selected miR-369-5p, miR-136 (which maps to 14q32; S1 Table) and miR-34a (mapping in 1p36.22), involved in senescence and tumor suppression [44,45] for quantitative RT-qPCR validation analysis. In independent batches of P_L_ cultured hADSCs, we confirmed that all three miRNAs were upregulated in 3% [O_2_], both in adult and pediatric ADSCs, as compared with P_S_ cultures (Fig 2B, S3 and S4 Figs). To exclude major alterations in the miRNA biogenesis machinery as a secondary cause of general miRNA upregulation in the 14q32 region during replicative senescence, we evaluated the expression of *DICER* after long-term cell culture periods at 21% or 3% [O_2_], and with both hADSCs populations, finding no significant reduction in its expression (S4C Fig). These findings strongly suggest a deregulated 14q32 miRNA expression pattern is associated with replicative senescence in P_L_ hADSC cultures.

### Deregulation of the 14q32 locus in long-term-cultured hADSCs is partially mediated by epigenetic modifications

Two main differentially methylated regions (DMRs) have been reported in the 14q32 locus: both intergenic (IG)-DMR and *MEG*-DMR (S1E Fig) can behave as imprinting control regions to regulate monoallelic expression of 14q32 locus clustered genes [46, 47]. To determine whether *MEG3* and miRNA upregulation are mediated by epigenetic modification *via* DNA methylation, we performed bisulfite-sequencing analysis on hADSCs grown at 3% [O_2_].

**Fig 3:**
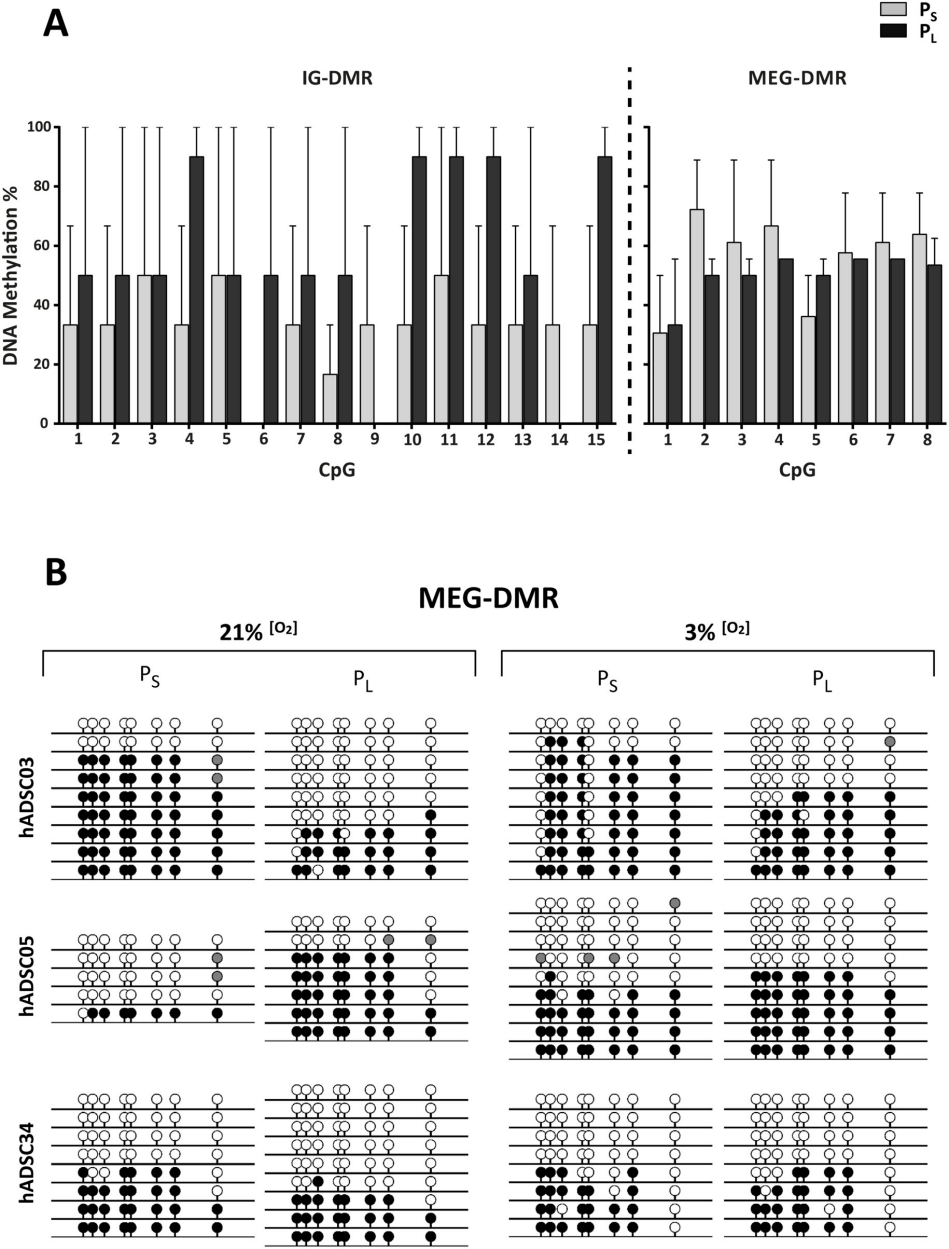
DNA methylation analysis of *DLK1-DIO3* IG- and *MEG*-DMR regions. (A) Percentage of DNA methylation in hADSC samples; x-axes indicate the CpG analyzed in the IG-DMR (1–15) and in the *MEG*-DMR region (1–8). Data represent mean ± SEM for P_S_ and P_L_ cultures. (B) Bisulfite genomic sequencing of the *MEG*-DMR regions. CpG dinucleotides are represented as lollipops; methylated cytosines, black; unmethylated cytosines, white. Cultures grown at 21% or 3% [O_2_] (P_S_ *vs*. P_L_) were compared for the indicated hADSC samples.

Surprisingly, the CpG methylation pattern of the individual clones analyzed to study IG-DMR showed considerable methylation heterogeneity (data not shown), which was unexpected for a DMR region. Moreover, the IG-DMR region showed no consistent differences in the hADSC samples under any experimental condition (P_L_/P_S_), even though there was a tendency for increased methylation with passages when the CpGs of the region were analyzed individually (Fig 3A).

In the *MEG*-DRM DNA methylation analysis, we observed a methylation pattern of the different analyzed clones consistent with those expected from a DMR region (Fig 3B). As was the case for IG-DMR, the analysis of the *MEG*-DMR region showed no significant P_L_/P_S_ differences in CpG methylation status in samples cultured to senescence (Fig 3A), even though individual CpG analysis showed a moderate tendency of decreased methylation with passages. Indeed, only the hADSC03 cell isolate cultured to senescence showed a clear DNA demethylation of the *MEG*-DMR region. To establish whether the effect observed in the *MEG3*-DMR was due to the oxygen tension, we repeated the DNA methylation analysis on hADSC samples cultured to senescence under 21% [O_2_]. In this case we observed similar results for *MEG3*-DMR to those observed in 3% [O_2_] cultures (Fig 3B).

**Fig 4:**
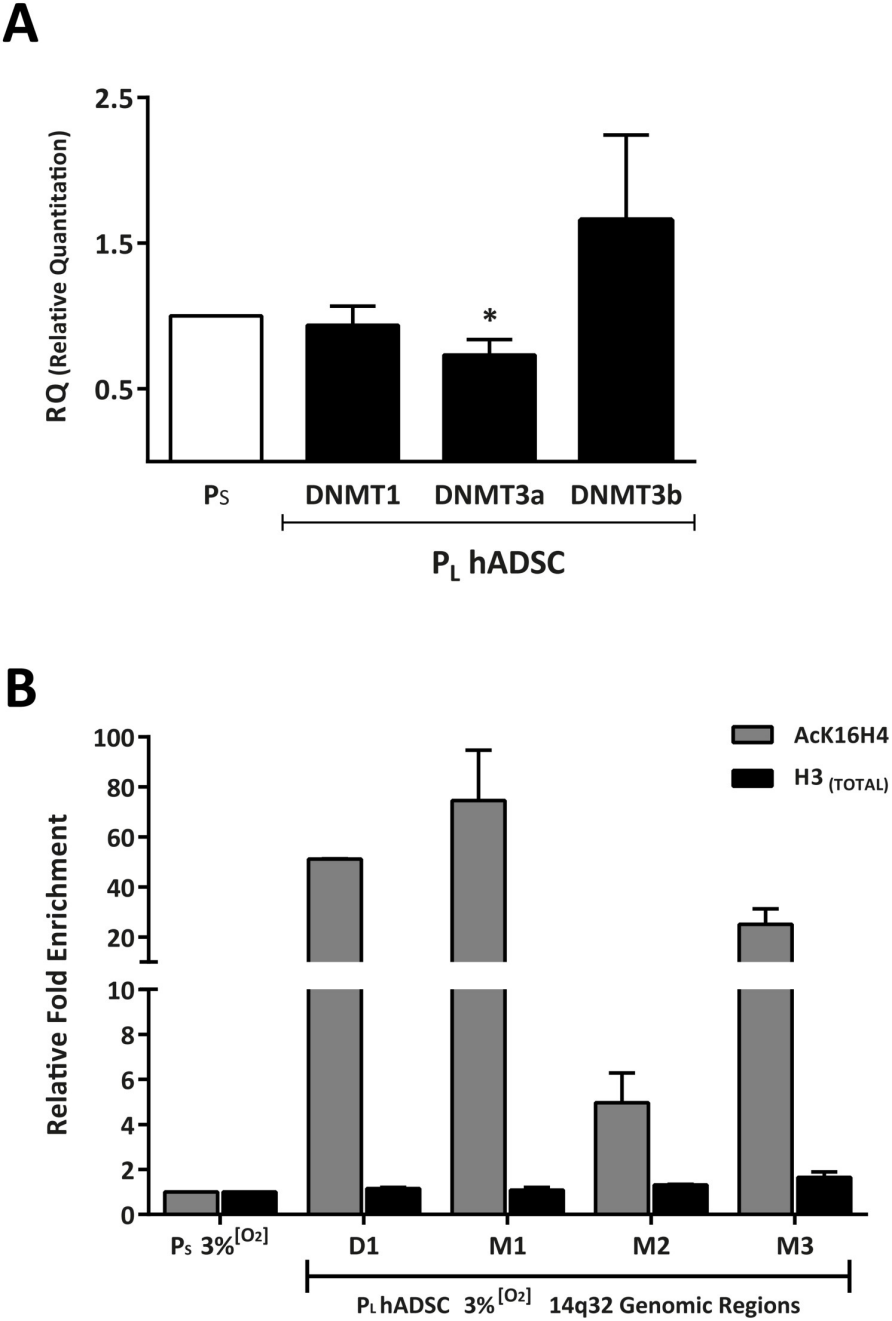
Epigenetic analysis of DLK1-DIO3 DMR regions. (A) Relative quantitation of *DNMT1*, *DNMT3a* and *DNMT3b* (hADSCs; *n* = 5 biological replicates). Data represent mean ± SEM (* p <0.05; two-tailed paired t-test). (B) Fold enrichment of AcK16H4 relative to total H3 in hADSC05 cultured at 3% [O_2_], comparing P_S_ and P_L_ samples. Enriched DNA was analyzed by qPCR using primers specific for the different regions (D1, M1–M3; see S1 Fig and S4 Table). Nonspecific adjustment (dCq) was calculated by (dCq = Cq_[IP]_ − Cq_[IgG]_). Fold enrichment was calculated as 2^(-ddCq) where ddCq is (ddCq = Cq_[PL]_ − Cq_[PS]_).

Analysis of the main enzymes of the methylation machinery (DNMT1, DNMT3a and DNMT3b) showed no significant modification in their gene expression, with the exception of *DNMT3a* whose expression was significantly decreased in P_L_ ADSC cultures (Fig 4A). Globally, although hADSCs treated with the DNMT inhibitor 5-aza-2’-deoxycytidine (AZA) demonstrated increased *MEG3* expression (S4 Fig), the results suggested no direct correlation between hADSC replicative senescence and DNA methylation in the analyzed regions for IG- and *MEG3*-DMR.

We then explored other epigenetic modifications potentially involved in the upregulation of the 14q32 locus non-coding genes. Treatment of hADSCs with trichostatin A (TSA), a histone deacetylase inhibitor, significantly increased *MEG3* expression both at P_L_ and P_S_ (S4D Fig), suggesting the involvement of histone deacetylation in regulating *MEG3* expression during senescence. We therefore carried out ChIP to study distribution of an activating acetylation mark (AcK16H4) in different regulatory sequences of the *DLK1-DIO3* locus, comparing P_L_ and P_S_ at 3% [O_2_]. The results showed that the acetylated K16H4 mark was enriched (30- to 75-fold) in P_L_ hADSCs, relative to P_S_ hADSCs, in most segments tested (D1, M1, M3); the M2 segment was also enriched, but at a lower level (Fig 4B). Segment D1 is upstream of *DLK1*-DMR, and segments M1–M3 are found in *MEG3* and upstream of the miRNA cluster (see S1 Fig). Overall, these findings suggest that the AcK16H4 activating mark is significantly associated with the upregulation of the entire *DLK1-DIO3* locus during hADSC replicative senescence.

## Discussion

The *DLK1-DIO3* imprinted locus has attracted much attention because it is one of the few loci critically altered during generation of murine and human induced pluripotent stem cells [48, 49]. Expression of coding and non-coding genes of the *Dlk1-Dio3* imprinted cluster (*Gtl2*/*Meg3*, miR-127, miR-134 and miR-154) is deregulated due to aberrant DNA methylation of the maternal allele, whereas the paternal allele preserves a normal methylation status [38]. Preservation of *Dlk1-Dio3* imprinting is improved by Dppa3/Pgc7 (developmental pluripotency-associated protein 3) binding to discrete domains of the IG-DMR region, and competing with Dnmt3a to preserve the original imprinting [49]; Polycomb Repressive Complex 2 (PRC2) is also a key element in maintaining expression of maternal miRNA and lncRNA from the *Dlk1-Dio3* locus [38]. More recently, it has been demonstrated that IG-DMR acts as an enhancer for the entire locus and that PRC2 interacts physically with Gtl2/Meg3 and Dnmt3 methyltransferase, reducing methylation at the IG-DMR [50]. Interestingly, it has also been recently reported that several ncRNAs corresponding to the *Gtl2*/*Meg3* domain, including *Gtl2*, are enriched in the murine hematopoietic stem cell pool (HSC; CD49b^lo^). These ncRNAs regulate activation of the PI3K-mTOR pathway and mutant HSCs (IG-DMR deletion) presented increased mitochondrial biogenesis, metabolic activity and ROS levels [51]. Therefore, it can be tentatively concluded that a balanced cell-specific expression of the *Dlk1-Dio3* locus is essential for optimal functional maintenance of pluripotent, fetal and adult stem cells.

Our work on replicative senescence in hADSCs revealed also an important role for the imprinted *DLK1-DIO3* locus. Several studies show that low oxygen concentrations (3% [O_2_]), significantly lower than atmospheric (21% [O_2_] pressure) improve the proliferation rate and quality of myriad tissue-derived cell cultures. In hADSCs, it was demonstrated that low oxygen concentrations reduce the senescence ratio during expansion [2–5]. Our results indicate that expanded hADSCs (both at 21 or 3% [O_2_], and using adult or pediatric isolates) present a progressive increase in *MEG3* expression, and this effect could be reverted by constitutive expression of the TERT catalytic subunit. *MEG3* has been proposed as a tumor suppressor and a negative regulator of angiogenesis [30,31], and its down-regulation serves as an unfavorable risk factor for survival in multiple human cancer types [32–34], mainly by promoter and IG-DMR region hypermethylation [52]. Interestingly, it has been proposed to also act as a competing endogenous RNA for several miRNAs (sponging), including miR-15a-5p, miR-19a, miR-664 and miR-214 [53]. In addition, it is interesting to note that trans-associations between the IGF2, MEG3 and DLK1 imprinted gene products have been proposed and that their three-dimensional nuclear organization is linked to the transcriptional state of these genes [54]. These findings suggest that during adult ADSC proliferative senescence, multiple imprinted loci could co-operatively be affected.

In addition, our miRNA analysis demonstrated that a quite significant fraction (69%) of senescence-upregulated miRNAs map also in the 14q32.31 cluster, involving miRNAs previously associated with senescence. For example, miR-136 is linked to fibroblast senescence, promoting apoptosis in glioma cells by targeting several anti-apoptotic genes [55], and miR-369-5p has previously been associated with hADSC senescence [13,21]. This means that the 14q32.31 miRNA cluster response is the major miRNA cellular response associated with replicative senescence. Comparison of our results with those of a previous study of radiation-induced senescence in hADSC [6] confirmed upregulation of some of the miRNAs identified here (e.g., miR-629-3p and miR-34a-5p). In agreement, *in silico* analysis of the putative target genes for senescence-deregulated miRNAs of the 14q32.31 locus (S2 Table; >1400 genes) highlighted biological functions involved in cell aging, gene expression, cell cycle and apoptosis (S3 Table). Several of these genes have been also identified as age-associated [56]. One of the clearest hallmarks of senescent hADSCs is a marked decrease in expression of AP-1 components (FOS and JUN, and their phosphorylated forms), which are critically associated with mobility and responsiveness limitation [6]. In the context discussed here, FOS is a significant target of miR-543 and miR-221, both up-regulated by senescence, and mapping in the 14q32.31 miRNA cluster. Finally, and in close agreement with the interpretation of our data, a recent study identified as tumor suppressors 26 targets for the entire 14q32.31 miRNA cluster [57].

The progressive and significant up-regulation of *MEG3* and 14q32.31 miRNA cluster (the largest described so far, with up to 54 described miRNAs) expression could have a significant pleiotropic effect, suppressing proliferation and promoting apoptosis/senescence of hADSCs. Comparing the evidence obtained in senescent hADSCs with that in reprograming schemes in adult cells [43–45] or in murine hematopoietic stems cells [46], it is clear that during replicative senescence of hADSCs, alterations in expression regulation are not dominated by a strong differential methylation of the main DMRs regulatory regions, although a compatible downregulation of *DMT3A* was found (Fig 4A). On the contrary, histone acetylation on specific domains seems to play a more decisive role. That said, a significant proportion of the miRNAs affected by IG-DMR deletion [51] are also found upregulated in senescent hADSCs (S1 Table), showing differences and similarities in those processes, all connected by the complex regulation of the imprinted *DLK1-DIO3* locus. During hADSC replicative senescence, there is progressive accumulation of *MEG3* expression that, based on the known regulatory features of the *Dlk1-Dio3* locus [44,49], could generate a feedback activation loop to promote and/or maintain IG-DMR overactivation. Given the central role proposed for *Gtl2*/*Meg3* as an enhancer of the *Dlk1-Dio3* locus, we speculate that these regulatory mechanisms could be altered during hADSC senescence, but not critically related to methylation.

## Conclusions

Our data strongly support a direct relationship between *DLK1-DIO3* (14q32) deregulation and replicative senescence of hADSCs. We found a specific profile that includes progressive upregulation of *DLK1-DIO3*-encoded lncRNA *MEG3* expression and upregulation of a very significant fraction of its largest miRNA cluster (14q32.31). Both of these main features are associated with tumor suppression and a putative correlation has been also proposed between *MEG3* upregulation and physiological aging and replicative senescence of stem and progenitor cells [58–60]. This is consistent with the idea that senescence is primarily a tumor suppressor mechanism and that progressive hyperactivation of the *DLK1-DIO3* cluster is thus likely a potent concerted tumor suppressor network. Altered expression of *MEG3* is not mainly associated with methylation levels in DMR domains, but to an increase in histone activation marks in discrete domains.

## Supporting information

S1 Fig Loss of hADSC biological properties in long-term culture. (A) Model of hADSC proliferation kinetics. From the cumulative population doubling data for different hADSC cultures, we obtained a curve fitted to a polynomic function. The various hADSC cultures were used to establish a mean proliferation curve for 150 days. Each dot represents the mean ± SEM of data obtained by modeling the different hADSC cultures. (B) Percentage of cells in cell cycle phase G_0_/G_1_ at different cell culture stages (hADSCs, *n* = 4 biological replicates); the graph shows the mean ± SEM of different cell cultures (* p <0.05; two-tailed paired t-test). (C) Representative FSC-A/SSC-A plot diagrams, representing cell size and complexity in P_S_ and P_L_ hADSC cultures. (D) Light microscopy image exampling senescent morphology changes and SA-ß-gal staining of senescent (Ps) cell cultures. (E) Scheme of the imprinted 14q32.2-14q32.31 locus, including the regions analyzed by bisulfite genomic sequencing (BS), and by ChIP of AcK16H4; coding genes are shown as black boxes, non-coding genes as gray boxes and miRNAs as triangles.
(TIF)

S2 Fig Analysis of replicative senescence on *IGF2* expression in hADSCs and v-myc immortalized NSCs. (A) Relative quantitation of *IGF2* in hADSCs (*n* = 4 biological replicates; obtained from Inbiobank) and in hADSCs overexpressing hTERT (+hTERT; *n* = 2 biological replicates). (B) Relative quantitation of *MEG3* in v-myc-immortalized human NPCs (*n* = 3 technical replicates); data represent mean ± SEM (* p <0.05, ** p <0.01, *** p <0.001; two-tailed paired t-test).
(TIF)

S3 Fig Analysis of adult hADSCs. (A) Scatter plot showing distribution of the VSN-invariant normalized intensity data for short-term (P_S_) and long-term cultured (P_L_) hADSC samples (*n* = 3 biological replicates). (B) Relative quantitation of selected miRNAs for array validation in the samples used for the array expression assay; hADSC P_S_ and P_L_ samples (*n* = 3 biological replicates). Cultures were grown at 3% [O_2_].
(TIF)

S4 Fig Analysis of pediatric hADSCs. (A) Bar graph showing array data analysis as log fold-change expression in pediatric hADSCs (mean ± SEM) of all miRNAs in the 14q32 chromosome region analyzed. (B) Relative quantitation of *DICER* in hADSCs (*n* = 5 biological replicates) and pediatric hADSCs (*n* = 9 biological replicates) expanded both at 3% and 21% [O_2_]. (C) Relative quantitation of lncRNA *MEG3* in pediatric hADSC samples (P_S_ and P_L_), cultured at 21% [O_2_], treated or not (control) with epigenetic drugs; gray bars indicate samples analyzed at 72 h after TSA or 5-AZA treatments and gray stippled bars correspond to samples treated with drugs for 72 h, washed, recultured and analyzed after an additional 96 h. Bars represent mean ± SEM (*n* = 2 technical replicates) * p <0.05; two-tailed ratio paired t-test.
(TIF)

S1 Table miRNAs differentially expressed in short-term *versus* long-term hADSC cultures. Upregulated miRNAs shadowed in pale green correspond to those described as upregulated in HSC (CD49b^lo^) [56], controlling PI3K-mTOR pathway.
(PDF)

S2 Table miRNA predicted targets. Table compiles 1,478 predicted targets (see Materials and methods) of the deregulated miRNAs identified in array experiments, indicating those mRNAs that are predicted as targets for multiple miRNAs.
(PDF)

S3 Table GO analysis of validated targets of *DLK1-DIO3*-upregulated miRNAs. (DOCX)

S4 Table List of primers used in the study. (TIF)

S1 File Garcia-Lopez et al. PlosOne_SUPP. Extended methods.
(DOCX)
