# Supplementary figures and images for: Deregulation of the imprinted DLK1-DIO3 locus ncRNAs is associated with replicative senescence of human adipose-derived stem cells
Source: PLoS One. 2018 Nov 5;13(11):e0206534. doi: 10.1371/journal.pone.0206534 (PMC6218046; doi:10.1371/journal.pone.0206534)

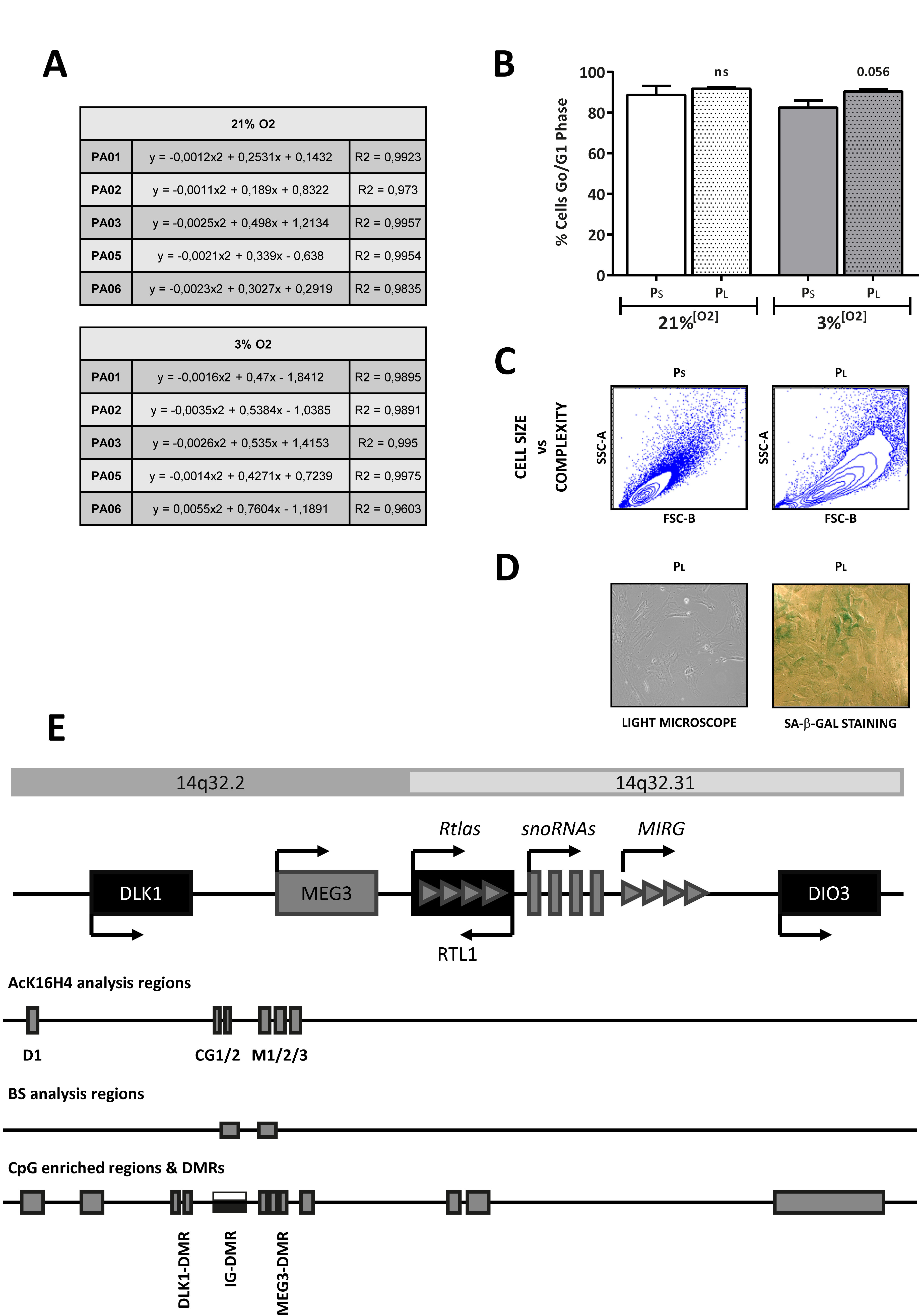

Supplement: S1 Fig — (A) Model of hADSC proliferation kinetics. From the cumulative population doubling data for different hADSC cultures, we obtained a curve fitted to a polynomic function. The various hADSC cultures were used to establish a mean proliferation curve for 150 days. Each dot represents the mean ± SEM of data obtained by modeling the different hADSC cultures. (B) Percentage of cells in cell cycle phase G0/G1 at different cell culture stages (hADSCs, n = 4 biological replicates); the graph shows the mean ± SEM of different cell cultures (* p <0.05; two-tailed paired t-test). (C) Representative FSC-A/SSC-A plot diagrams, representing cell size and complexity in PS and PL hADSC cultures. (D) Light microscopy image exampling senescent morphology changes and SA-ß-gal staining of senescent (Ps) cell cultures. (E) Scheme of the imprinted 14q32.2-14q32.31 locus, including the regions analyzed by bisulfite genomic sequencing (BS), and by ChIP of AcK16H4; coding genes are shown as black boxes, non-coding genes as gray boxes and miRNAs as triangles. (TIF) [file pone.0206534.s001.tif]

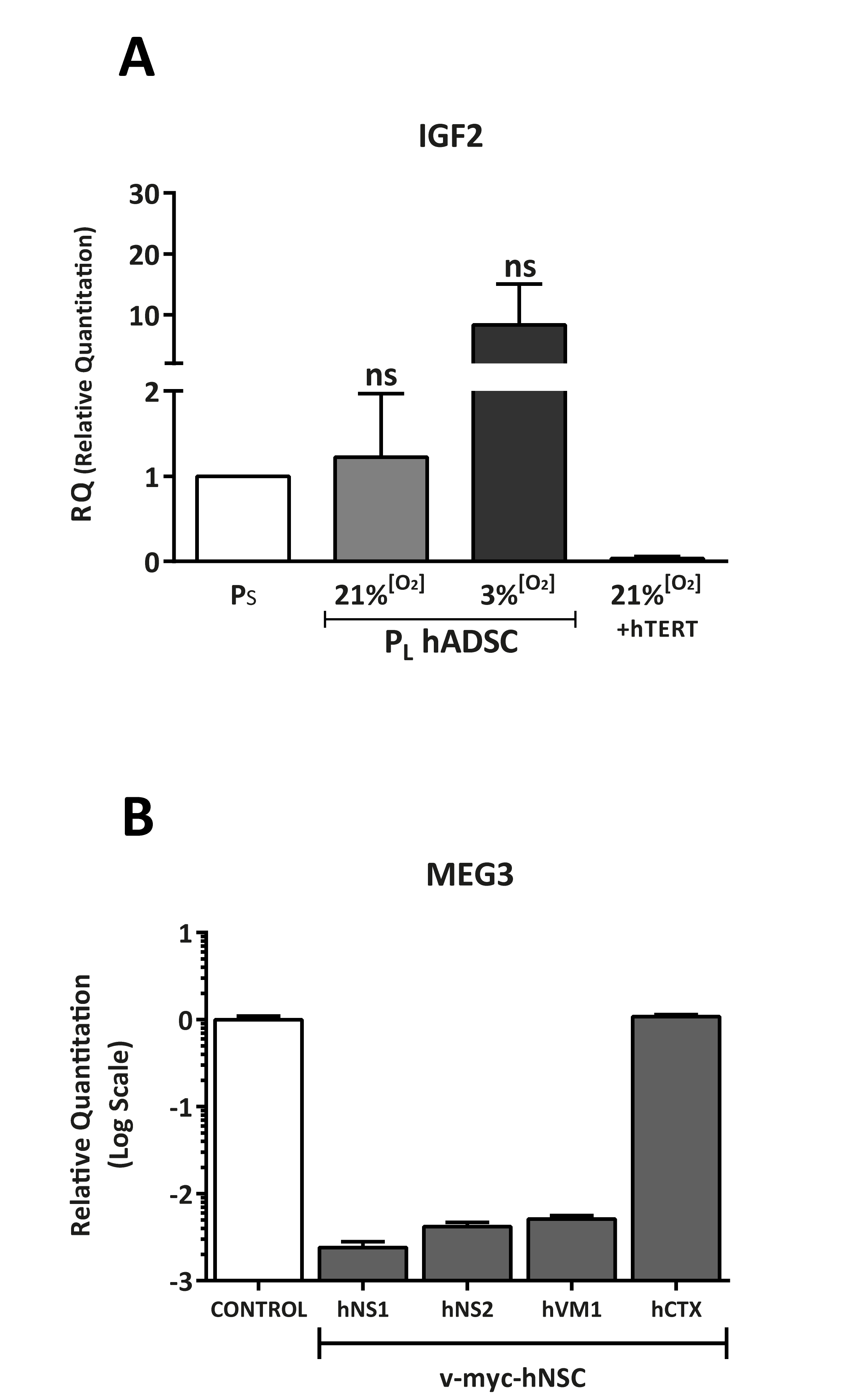

Supplement: S2 Fig — (A) Relative quantitation of IGF2 in hADSCs (n = 4 biological replicates; obtained from Inbiobank) and in hADSCs overexpressing hTERT (+hTERT; n = 2 biological replicates). (B) Relative quantitation of MEG3 in v-myc-immortalized human NPCs (n = 3 technical replicates); data represent mean ± SEM (* p <0.05, ** p <0.01, *** p <0.001; two-tailed paired t-test). (TIF) [file pone.0206534.s002.tif]

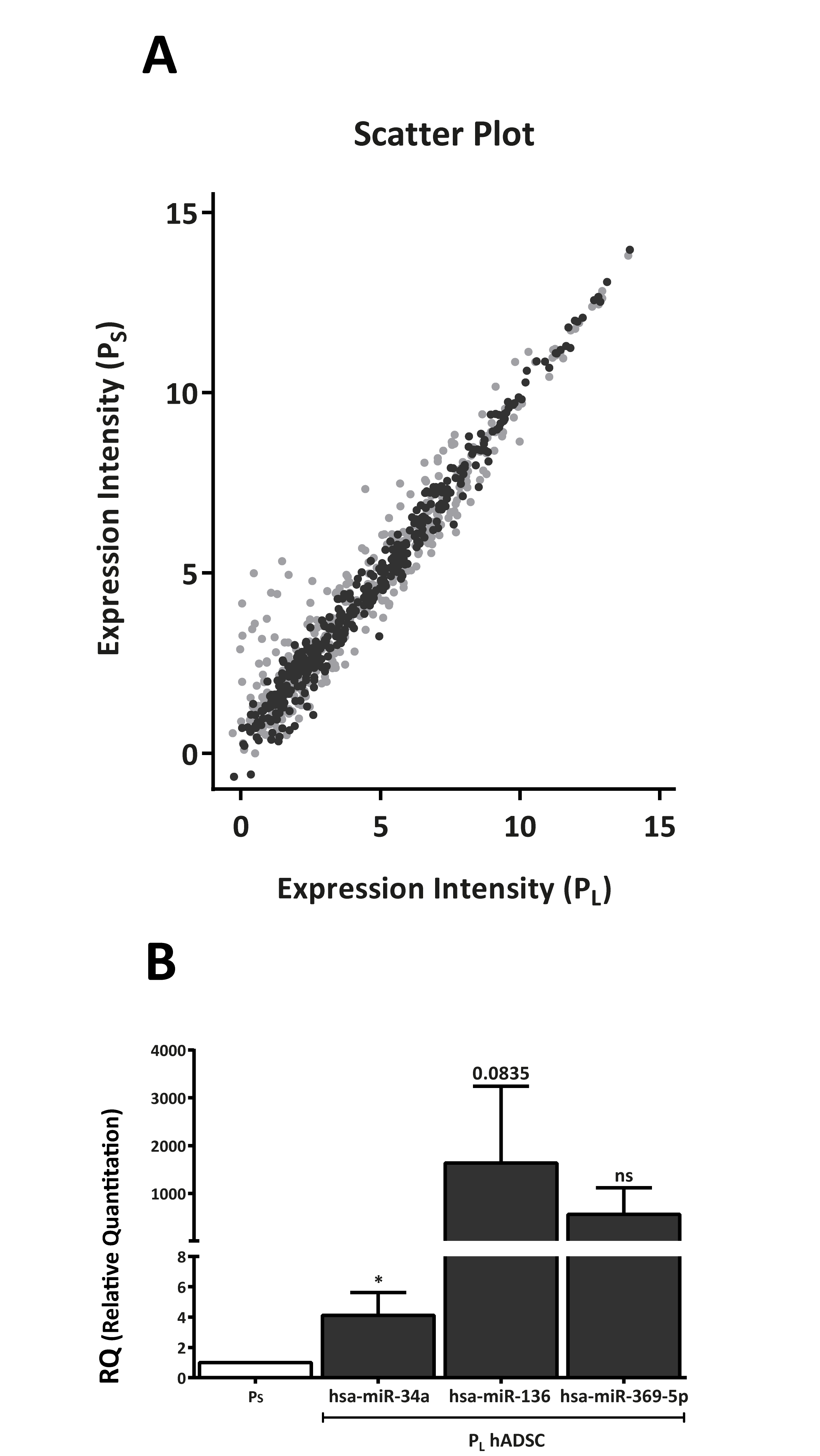

Supplement: S3 Fig — (A) Scatter plot showing distribution of the VSN-invariant normalized intensity data for short-term (PS) and long-term cultured (PL) hADSC samples (n = 3 biological replicates). (B) Relative quantitation of selected miRNAs for array validation in the samples used for the array expression assay; hADSC PS and PL samples (n = 3 biological replicates). Cultures were grown at 3% [O2]. (TIF) [file pone.0206534.s003.tif]

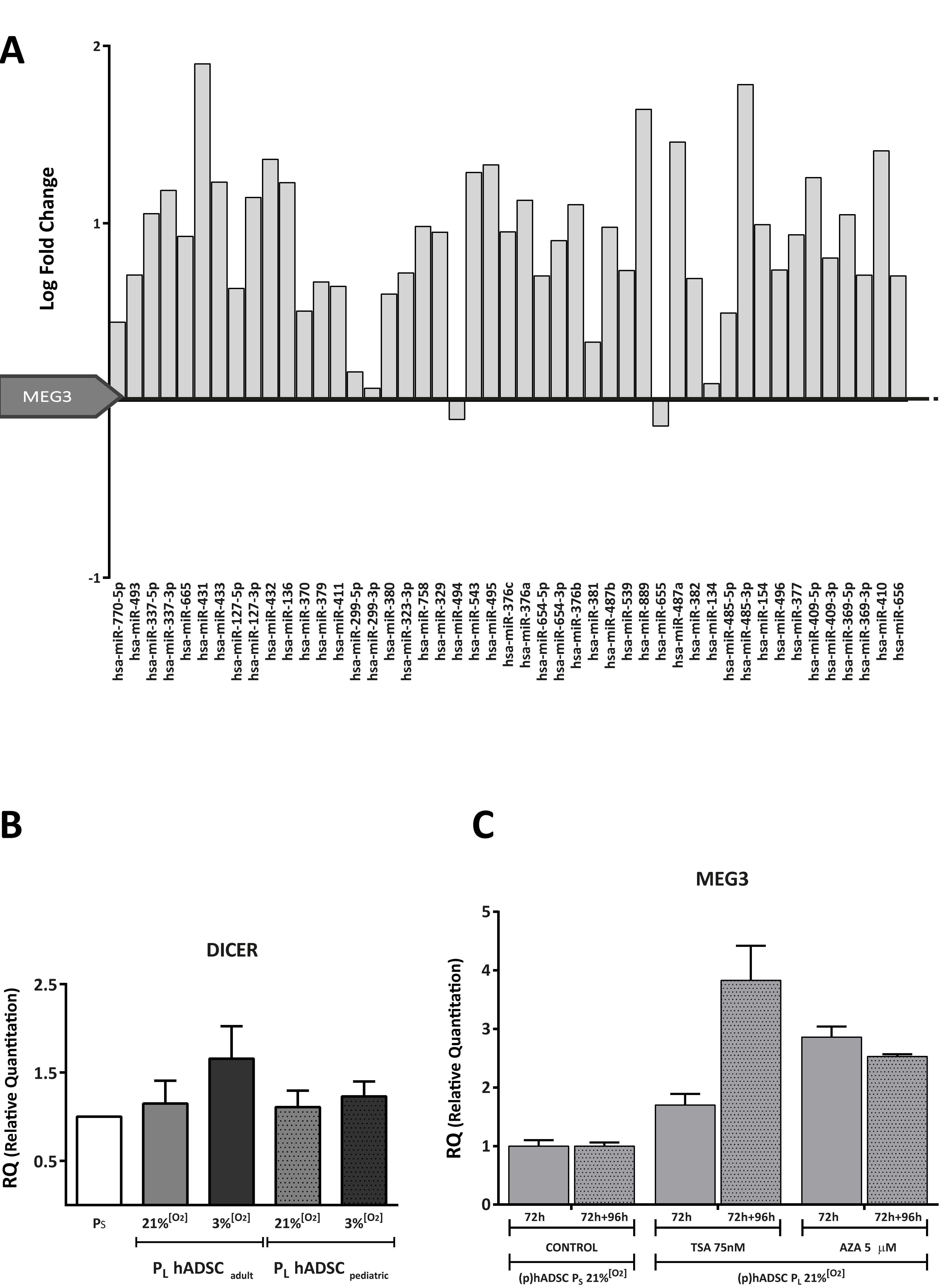

Supplement: S4 Fig — (A) Bar graph showing array data analysis as log fold-change expression in pediatric hADSCs (mean ± SEM) of all miRNAs in the 14q32 chromosome region analyzed. (B) Relative quantitation of DICER in hADSCs (n = 5 biological replicates) and pediatric hADSCs (n = 9 biological replicates) expanded both at 3% and 21% [O2]. (C) Relative quantitation of lncRNA MEG3 in pediatric hADSC samples (PS and PL), cultured at 21% [O2], treated or not (control) with epigenetic drugs; gray bars indicate samples analyzed at 72 h after TSA or 5-AZA treatments and gray stippled bars correspond to samples treated with drugs for 72 h, washed, recultured and analyzed after an additional 96 h. Bars represent mean ± SEM (n = 2 technical replicates) * p <0.05; two-tailed ratio paired t-test. (TIF) [file pone.0206534.s004.tif]

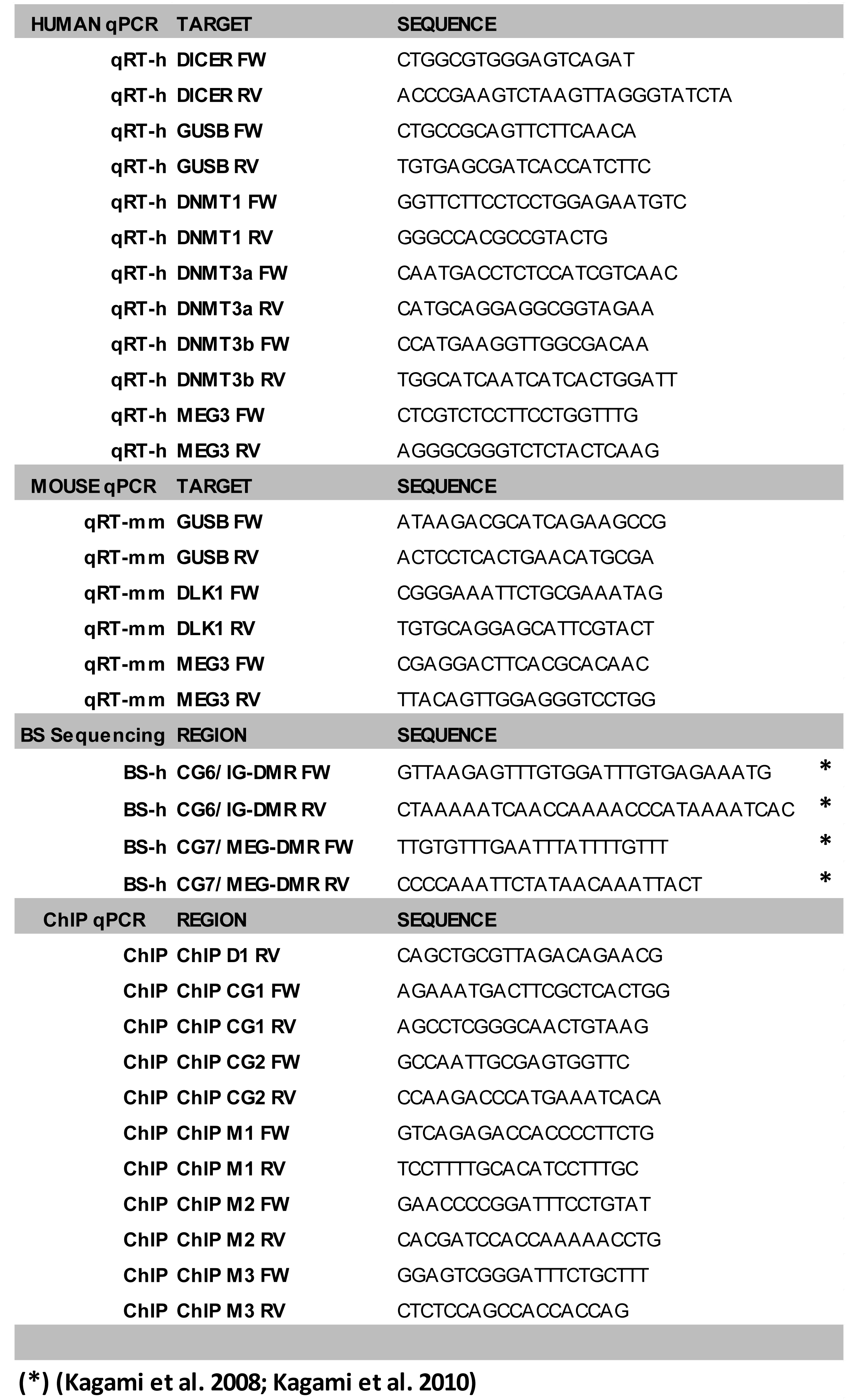

Supplement: S4 Table — (TIF) [file pone.0206534.s008.tif]
