# Supplementary material for: Deregulation of the imprinted DLK1-DIO3 locus ncRNAs is associated with replicative senescence of human adipose-derived stem cells
Source: PLoS One. 2018 Nov 5;13(11):e0206534. doi: 10.1371/journal.pone.0206534 (PMC6218046; doi:10.1371/journal.pone.0206534)
Supplement: S1 Table — Upregulated miRNAs shadowed in pale green correspond to those described as upregulated in HSC (CD49blo) [56], controlling PI3K-mTOR pathway. (PDF) [file pone.0206534.s005.pdf]

Probe	logFC	A	pvalue	adjpvalue	Location
hsa-miR-32*	-2,59	3,11	0,02	0,13	chr9:110848332-110848350
hsa-miR-885-5p	-2,44	2,19	0,01	0,12	chr3:010411215-010411230
hsa-miR-766	-2,27	3,63	0,02	0,12	chrX:118664754-118664767
hsa-miR-1224-3p	-2,05	1,85	0,00	0,12	chr3:185441971-185441956
hsa-miR-328	-2,01	3,02	0,02	0,12	chr16:065793731-065793747
hsa-miR-483-3p	-1,98	3,09	0,01	0,12	chr11:002111948-002111964
hsa-miR-877*	-1,95	2,87	0,01	0,12	chr6:030660173-030660161
hsa-miR-1227	-1,92	1,92	0,01	0,12	chr19:002185061-002185074
hsa-miR-937	-1,92	1,59	0,01	0,12	chr8:144967129-144967144
hsa-miR-647	-1,82	1,56	0,01	0,12	chr20:062044488-062044502
hsa-miR-595	-1,66	2,30	0,02	0,13	chr7:158018186-158018201
hsa-miR-197	-1,65	6,08	0,02	0,12	chr1:109943106-109943091
hsa-miR-1228*	-1,49	1,68	0,02	0,12	chr12:055874574-055874563
hsa-miR-19b-1*	-1,44	1,51	0,00	0,12	chr13:090801484-090801471
hsa-miR-195*	-1,23	1,25	0,01	0,12	chr17:006861671-006861683
hsa-let-7b*	-1,22	2,27	0,02	0,12	chr22:044888310-044888296
hsa-miR-491-3p	-1,15	1,81	0,04	0,19	chr9:020706174-020706155
hsa-miR-16-2*	-1,14	3,46	0,00	0,12	chr3:161605300-161605282
hsa-miR-744*	-1,10	2,04	0,00	0,12	chr17:011926029-011926012
hsa-miR-574-3p	-1,08	6,78	0,02	0,12	chr4:038546129-038546114
hsa-miR-625*	-1,05	1,62	0,00	0,12	chr14:065007645-065007627
hsa-let-7d*	-1,03	1,92	0,03	0,15	chr9:095981019-095981002
hsa-miR-34b	-1,03	3,29	0,01	0,12	chr11:110888943-110888928
hsa-miR-20a*	-1,01	1,61	0,02	0,12	chr13:090801384-090801365
hsa-miR-15b*	-1,00	2,09	0,01	0,12	chr3:161605148-161605127
hsa-miR-93*	-0,89	0,85	0,00	0,12	chr7:099529336-099529351
hsa-miR-106a	-0,81	3,65	0,01	0,12	chrX:133131940-133131952
hsa-miR-550*	-0,80	2,68	0,01	0,12	chr7:030296016-030296001
hsa-miR-17	-0,79	7,21	0,02	0,14	chr13:090800895-090800881
hsa-miR-20b	-0,79	6,06	0,01	0,12	chrX:133131546-133131564
hsa-miR-20a	-0,79	7,98	0,02	0,13	chr13:090801349-090801332
hsa-miR-92a	-0,76	7,05	0,01	0,12	chr13:090801637-090801623
hsa-miR-25	-0,76	7,45	0,02	0,12	chr7:099529130-099529146
hsa-miR-93	-0,74	7,41	0,03	0,16	chr7:099529374-099529389
hsa-miR-7-1*	-0,73	2,99	0,02	0,13	chr9:085774506-085774524
hsa-miR-374b*	-0,72	1,12	0,01	0,12	chrX:073355117-073355138
hsa-miR-30b*	-0,71	1,79	0,01	0,12	chr8:135881957-135881975
hsa-miR-671-3p	-0,71	0,47	0,01	0,12	chr7:150566527-150566518
hsa-miR-16	-0,70	10,51	0,01	0,12	chr13:049521164-049521181
hsa-miR-629	-0,68	1,67	0,03	0,16	chr15:068158820-068158834
hsa-miR-15b	-0,67	9,48	0,03	0,15	chr3:161605110-161605091
hsa-miR-15a	-0,64	8,42	0,02	0,13	chr13:049521304-049521324
hsa-miR-221	0,49	10,85	0,02	0,14	chrX:045490552-045490570
hsa-miR-654-3p	0,54	7,03	0,03	0,15	chr14:100576380-100576363
hsa-miR-181a	0,55	5,89	0,01	0,12	chr1:197094860-197094873
hsa-miR-379	0,55	6,28	0,03	0,15	chr14:100558181-100558167
hsa-miR-152	0,55	7,11	0,03	0,15	chr17:043469539-043469554
hsa-miR-487b	0,56	6,97	0,02	0,14	chr14:100582616-100582599
hsa-miR-154*	0,56	6,21	0,03	0,15	chr14:100595916-100595897
hsa-miR-411	0,58	5,54	0,02	0,13	chr14:100559450-100559433
hsa-miR-665	0,59	1,97	0,04	0,18	chr14:100411184-100411172
hsa-miR-24-1*	0,61	3,16	0,03	0,15	chr9:096888151-096888131
hsa-miR-377*	0,61	2,63	0,02	0,12	chr14:100598167-100598149
hsa-miR-376c	0,65	8,57	0,01	0,12	chr14:100575842-100575824
hsa-miR-543	0,66	5,53	0,01	0,12	chr14:100568144-100568126
hsa-miR-493*	0,67	6,94	0,01	0,12	chr14:100405186-100405168

hsa-miR-601	0,67	1,91	0,01	0,12	chr9:125204667-125204682
hsa-miR-432	0,69	5,20	0,00	0,12	chr14:100420608-100420591
hsa-miR-337-3p	0,70	5,43	0,01	0,12	chr14:100410664-100410644
hsa-miR-370	0,70	4,50	0,01	0,12	chr14:100447297-100447284
hsa-miR-409-3p	0,71	6,96	0,01	0,12	chr14:100601457-100601443
hsa-miR-377	0,71	8,22	0,01	0,12	chr14:100598205-100598187
hsa-miR-193a-3p	0,72	8,19	0,01	0,12	chr17:026911203-026911187
hsa-miR-149*	0,73	0,77	0,01	0,12	chr2:241044166-241044154
hsa-miR-410	0,73	5,66	0,01	0,12	chr14:100602071-100602053
hsa-miR-127-3p	0,74	7,81	0,02	0,14	chr14:100419146-100419129
hsa-miR-498	0,75	1,70	0,02	0,12	chr19:058869318-058869303
hsa-miR-34a	0,77	9,28	0,03	0,15	chr1:009134381-009134399
hsa-miR-654-5p	0,78	2,93	0,02	0,12	chr14:100576345-100576329
hsa-miR-376a	0,78	8,48	0,01	0,12	chr14:100576228-100576211
hsa-miR-134	0,78	5,28	0,03	0,15	chr14:100590805-100590794
hsa-miR-409-5p	0,79	4,57	0,00	0,12	chr14:100601426-100601410
hsa-miR-369-5p	0,79	4,69	0,01	0,12	chr14:100601717-100601698
hsa-miR-369-3p	0,79	2,84	0,00	0,12	chr14:100601751-100601731
hsa-miR-136*	0,82	5,57	0,00	0,12	chr14:100420861-100420842
hsa-miR-495	0,83	6,69	0,00	0,12	chr14:100569915-100569896
hsa-miR-154	0,84	5,38	0,01	0,12	chr14:100595880-100595863
hsa-miR-373*	0,87	1,61	0,04	0,19	chr19:058983797-058983785
hsa-miR-337-5p	0,88	6,63	0,00	0,12	chr14:100410625-100410609
hsa-miR-663	0,92	3,34	0,01	0,12	chr20:026136879-026136891
hsa-miR-1225-5p	0,95	6,78	0,03	0,15	chr16:002080265-002080274
hsa-miR-376a*	1,00	5,28	0,00	0,12	chr14:100576899-100576879
hsa-miR-136	1,22	7,17	0,00	0,06	chr14:100420828-100420806
hsa-miR-126	1,23	2,37	0,01	0,12	chr9:138684947-138684931
