# Supplementary material for: Deregulation of the imprinted DLK1-DIO3 locus ncRNAs is associated with replicative senescence of human adipose-derived stem cells
Source: PLoS One. 2018 Nov 5;13(11):e0206534. doi: 10.1371/journal.pone.0206534 (PMC6218046; doi:10.1371/journal.pone.0206534)
Supplement: S2 Table — Table compiles 1,478 predicted targets (see Materials and methods) of the deregulated miRNAs identified in array experiments, indicating those mRNAs that are predicted as targets for multiple miRNAs. (PDF) [file pone.0206534.s006.pdf]

miRNum	Target	miRNA
9	WEE1	hsa-miR-106a-5p hsa-miR-15a-5p hsa-miR-15b-3p hsa-miR-15b-5p hsa-miR-16-5p hsa-miR-20a-5p hsa-miR-221-3p hsa-miR-374b-3p hsa-miR-877-3p
8	BTG2	hsa-miR-15a-5p hsa-miR-15b-5p hsa-miR-16-5p hsa-miR-17-3p hsa-miR-20a-5p hsa-miR-25-3p hsa-miR-34b-5p hsa-miR-92a-3p
8	NUCKS1	hsa-miR-15a-5p hsa-miR-15b-5p hsa-miR-16-2-3p hsa-miR-16-5p hsa-miR-25-3p hsa-miR-877-3p hsa-miR-92a-3p hsa-miR-93-3p
8	NUFIP2	hsa-miR-106a-5p hsa-miR-15a-5p hsa-miR-15b-5p hsa-miR-16-5p hsa-miR-193a-3p hsa-miR-20a-5p hsa-miR-25-3p hsa-miR-92a-3p
7	E2F3	hsa-miR-15a-5p hsa-miR-15b-5p hsa-miR-16-5p hsa-miR-17-5p hsa-miR-20a-5p hsa-miR-34a-5p hsa-miR-92a-3p
7	HBP1	hsa-let-7b-3p hsa-miR-106a-5p hsa-miR-17-3p hsa-miR-17-5p hsa-miR-20a-5p hsa-miR-20b-5p hsa-miR-766-5p
7	KLHL15	hsa-miR-15a-5p hsa-miR-15b-5p hsa-miR-16-2-3p hsa-miR-16-5p hsa-miR-20a-5p hsa-miR-25-3p hsa-miR-92a-3p
6	CCND1	hsa-miR-106a-5p hsa-miR-15a-5p hsa-miR-15b-5p hsa-miR-16-5p hsa-miR-20a-5p hsa-miR-34a-5p
6	DDX3X	hsa-miR-106a-3p hsa-miR-15a-5p hsa-miR-15b-5p hsa-miR-16-5p hsa-miR-25-3p hsa-miR-92a-3p
6	EIF4G2	hsa-miR-106a-5p hsa-miR-17-5p hsa-miR-20a-5p hsa-miR-20b-5p hsa-miR-25-3p hsa-miR-92a-3p
6	MIDN	hsa-miR-106a-5p hsa-miR-17-5p hsa-miR-20a-5p hsa-miR-20b-5p hsa-miR-221-3p hsa-miR-766-5p
6	MYLIP	hsa-miR-106a-5p hsa-miR-20a-5p hsa-miR-20b-5p hsa-miR-221-3p hsa-miR-25-3p hsa-miR-92a-3p
6	PTEN	hsa-miR-17-5p hsa-miR-20a-5p hsa-miR-20b-5p hsa-miR-221-3p hsa-miR-543 hsa-miR-92a-3p
6	SIK1	hsa-miR-106a-5p hsa-miR-15a-5p hsa-miR-15b-5p hsa-miR-16-5p hsa-miR-20a-5p hsa-miR-92a-3p
6	SKI	hsa-miR-15a-5p hsa-miR-15b-5p hsa-miR-16-5p hsa-miR-20a-5p hsa-miR-374b-3p hsa-miR-92a-3p
6	SMAD7	hsa-miR-15a-5p hsa-miR-15b-5p hsa-miR-16-5p hsa-miR-25-3p hsa-miR-374b-3p hsa-miR-766-5p
6	SRPR	hsa-miR-15a-5p hsa-miR-15b-5p hsa-miR-16-5p hsa-miR-25-3p hsa-miR-374b-3p hsa-miR-92a-3p
6	ZBTB5	hsa-miR-106a-5p hsa-miR-15a-5p hsa-miR-15b-5p hsa-miR-16-5p hsa-miR-20a-5p hsa-miR-221-3p
5	CCND2	hsa-miR-15a-5p hsa-miR-15b-5p hsa-miR-16-5p hsa-miR-17-5p hsa-miR-20a-5p
5	CCNE2	hsa-miR-15a-5p hsa-miR-15b-5p hsa-miR-16-5p hsa-miR-25-3p hsa-miR-34a-5p
5	CDK6	hsa-miR-15a-5p hsa-miR-15b-5p hsa-miR-16-5p hsa-miR-221-3p hsa-miR-34a-5p
5	ELK4	hsa-miR-106a-5p hsa-miR-15a-5p hsa-miR-15b-5p hsa-miR-16-5p hsa-miR-20a-5p
5	EZH1	hsa-miR-15a-5p hsa-miR-15b-5p hsa-miR-16-5p hsa-miR-20a-5p hsa-miR-766-3p
5	FEM1C	hsa-miR-106a-5p hsa-miR-16-5p hsa-miR-17-5p hsa-miR-20a-5p hsa-miR-20b-5p
5	KATNAL1	hsa-miR-106a-5p hsa-miR-15a-5p hsa-miR-15b-5p hsa-miR-16-5p hsa-miR-20a-5p

5	KIF5B	hsa-miR-15a-5p hsa-miR-15b-5p hsa-miR-16-5p hsa-miR-25-3p hsa-miR-92a-3p
5	NOTCH2	hsa-miR-15a-5p hsa-miR-15b-5p hsa-miR-16-5p hsa-miR-181a-5p hsa-miR-34a-5p
5	NUP50	hsa-miR-15a-5p hsa-miR-15b-5p hsa-miR-16-5p hsa-miR-34a-3p hsa-miR-877-3p
5	RPRD2	hsa-miR-106a-5p hsa-miR-15a-5p hsa-miR-15b-5p hsa-miR-16-5p hsa-miR-30b-3p
5	SGMS1	hsa-miR-106a-5p hsa-miR-17-5p hsa-miR-20a-5p hsa-miR-20b-5p hsa-miR-32-3p
5	STAT3	hsa-miR-106a-5p hsa-miR-17-5p hsa-miR-181a-5p hsa-miR-20a-5p hsa-miR-20b-5p
5	SUPT16H	hsa-miR-15a-5p hsa-miR-15b-5p hsa-miR-16-5p hsa-miR-647 hsa-miR-877-3p
5	SUV420H1	hsa-miR-106a-5p hsa-miR-17-5p hsa-miR-20a-5p hsa-miR-20b-5p hsa-miR-92a-3p
5	ULK1	hsa-miR-106a-5p hsa-miR-17-5p hsa-miR-181a-5p hsa-miR-20a-5p hsa-miR-20b-5p
5	ZBTB33	hsa-miR-106a-5p hsa-miR-15a-5p hsa-miR-15b-5p hsa-miR-16-5p hsa-miR-20a-5p
5	ZMAT3	hsa-miR-15a-5p hsa-miR-15b-5p hsa-miR-16-5p hsa-miR-193a-3p hsa-miR-374b-3p
4	AGO4	hsa-miR-15a-5p hsa-miR-15b-5p hsa-miR-16-5p hsa-miR-92a-3p
4	ABL2	hsa-miR-15a-5p hsa-miR-15b-5p hsa-miR-16-5p hsa-miR-20a-5p
4	ACTR2	hsa-miR-15a-5p hsa-miR-15b-5p hsa-miR-16-5p hsa-miR-20a-5p
4	AFF4	hsa-miR-15a-5p hsa-miR-15b-5p hsa-miR-16-5p hsa-miR-181a-5p
4	AKAP11	hsa-miR-15a-5p hsa-miR-15b-5p hsa-miR-16-5p hsa-miR-20a-5p
4	ANKFY1	hsa-miR-193a-3p hsa-miR-20a-5p hsa-miR-30b-3p hsa-miR-766-3p
4	ANKRD13C	hsa-miR-17-5p hsa-miR-181a-5p hsa-miR-20a-5p hsa-miR-20b-5p
4	ARCN1	hsa-miR-15a-5p hsa-miR-15b-5p hsa-miR-16-5p hsa-miR-20a-5p
4	ASH1L	hsa-miR-15a-5p hsa-miR-15b-5p hsa-miR-16-5p hsa-miR-374b-3p
4	ATL3	hsa-miR-106a-5p hsa-miR-17-5p hsa-miR-20a-5p hsa-miR-20b-5p
4	ATP13A3	hsa-miR-15a-5p hsa-miR-15b-5p hsa-miR-16-5p hsa-miR-34a-3p
4	ATXN1	hsa-miR-106a-5p hsa-miR-20a-5p hsa-miR-221-3p hsa-miR-92a-3p
4	ATXN7L3B	hsa-miR-15a-5p hsa-miR-15b-5p hsa-miR-16-5p hsa-miR-20a-5p
4	BCL2	hsa-miR-15a-5p hsa-miR-15b-5p hsa-miR-16-5p hsa-miR-34a-5p
4	BICD2	hsa-miR-106a-5p hsa-miR-17-5p hsa-miR-20a-5p hsa-miR-766-3p
4	BNIP2	hsa-miR-106a-5p hsa-miR-17-5p hsa-miR-20a-5p hsa-miR-20b-5p
4	BZW1	hsa-miR-15a-5p hsa-miR-15b-5p hsa-miR-16-5p hsa-miR-19b-1-5p
4	CANX	hsa-miR-136-3p hsa-miR-15a-5p hsa-miR-15b-5p hsa-miR-16-5p
4	CAPZA2	hsa-miR-15a-5p hsa-miR-15b-5p hsa-miR-16-5p hsa-miR-32-3p
4	CCDC71L	hsa-miR-106a-5p hsa-miR-17-5p hsa-miR-20a-5p hsa-miR-20b-5p
4	CCNT1	hsa-miR-15a-5p hsa-miR-15b-5p hsa-miR-16-5p hsa-miR-221-5p
4	CD2AP	hsa-miR-15a-5p hsa-miR-15b-5p hsa-miR-16-5p hsa-miR-92a-3p
4	CDC27	hsa-miR-15a-5p hsa-miR-15b-5p hsa-miR-16-5p hsa-miR-92a-3p
4	CDKN1A	hsa-miR-106a-5p hsa-miR-17-5p hsa-miR-20a-5p hsa-miR-20b-5p
4	CRKL	hsa-miR-15a-5p hsa-miR-15b-5p hsa-miR-16-5p hsa-miR-17-3p
4	DCTN5	hsa-miR-15a-5p hsa-miR-15b-5p hsa-miR-16-5p hsa-miR-34a-5p
4	DNAJB9	hsa-miR-106a-5p hsa-miR-20a-5p hsa-miR-25-3p hsa-miR-92a-3p
4	DNAJC10	hsa-miR-15a-5p hsa-miR-15b-5p hsa-miR-16-5p hsa-miR-20a-5p
4	EFCAB14	hsa-miR-106a-5p hsa-miR-17-5p hsa-miR-20a-5p hsa-miR-20b-5p
4	EIF2S1	hsa-miR-106a-5p hsa-miR-17-5p hsa-miR-20a-5p hsa-miR-20b-5p

4	EN2	hsa-miR-15a-5p hsa-miR-15b-5p hsa-miR-16-5p hsa-miR-766-5p
4	FAM129A	hsa-miR-106a-5p hsa-miR-20a-5p hsa-miR-25-3p hsa-miR-92a-3p
4	FEM1B	hsa-miR-106a-5p hsa-miR-20a-5p hsa-miR-374b-3p hsa-miR-550a-3p
4	FGF2	hsa-miR-15a-5p hsa-miR-15b-5p hsa-miR-16-5p hsa-miR-92a-3p
4	FOXK1	hsa-miR-15a-5p hsa-miR-15b-5p hsa-miR-16-5p hsa-miR-17-3p
4	GALNT7	hsa-miR-16-5p hsa-miR-17-3p hsa-miR-25-3p hsa-miR-92a-3p
4	GATA6	hsa-miR-181a-5p hsa-miR-20a-5p hsa-miR-25-3p hsa-miR-92a-3p
4	GIGYF1	hsa-miR-106a-5p hsa-miR-20a-5p hsa-miR-34b-5p hsa-miR-877-3p
4	HDGF	hsa-miR-15a-3p hsa-miR-15a-5p hsa-miR-15b-5p hsa-miR-16-5p
4	HOXA10	hsa-miR-15a-5p hsa-miR-15b-5p hsa-miR-16-5p hsa-miR-411-3p
4	HSPA1B	hsa-miR-15a-5p hsa-miR-15b-5p hsa-miR-16-5p hsa-miR-34a-5p
4	ICMT	hsa-miR-106a-5p hsa-miR-17-5p hsa-miR-20a-5p hsa-miR-20b-5p
4	JARID2	hsa-miR-15a-5p hsa-miR-15b-5p hsa-miR-16-5p hsa-miR-197-3p
4	KPNA1	hsa-miR-15a-5p hsa-miR-15b-5p hsa-miR-16-5p hsa-miR-34a-5p
4	LAPTM4A	hsa-miR-106a-5p hsa-miR-17-5p hsa-miR-20a-5p hsa-miR-20b-5p
4	MORF4L1	hsa-miR-106a-5p hsa-miR-16-5p hsa-miR-17-5p hsa-miR-20a-5p
4	MYH9	hsa-miR-149-3p hsa-miR-30b-3p hsa-miR-877-3p hsa-miR-92a-3p
4	MYO5A	hsa-miR-15a-5p hsa-miR-15b-5p hsa-miR-16-5p hsa-miR-92a-3p
4	PAFAH1B1	hsa-miR-15a-5p hsa-miR-15b-5p hsa-miR-16-5p hsa-miR-20a-5p
4	PAFAH1B2	hsa-miR-15a-5p hsa-miR-15b-5p hsa-miR-16-5p hsa-miR-221-3p
4	PPP2R5C	hsa-miR-15a-5p hsa-miR-15b-5p hsa-miR-16-5p hsa-miR-193a-3p
4	PRKAR2A	hsa-miR-15a-5p hsa-miR-15b-5p hsa-miR-16-5p hsa-miR-197-3p
4	PROSC	hsa-miR-15a-5p hsa-miR-15b-5p hsa-miR-16-5p hsa-miR-34a-5p
4	PTAR1	hsa-miR-16-2-3p hsa-miR-25-3p hsa-miR-34b-5p hsa-miR-92a-3p
4	PTP4A1	hsa-miR-106a-5p hsa-miR-20a-5p hsa-miR-32-3p hsa-miR-374b-3p
4	PURA	hsa-miR-15a-5p hsa-miR-15b-5p hsa-miR-16-5p hsa-miR-20a-5p
4	RGMB	hsa-miR-106a-5p hsa-miR-17-5p hsa-miR-20a-5p hsa-miR-20b-5p
4	RORA	hsa-miR-106a-5p hsa-miR-20a-5p hsa-miR-32-3p hsa-miR-92a-3p
4	SESN3	hsa-miR-17-5p hsa-miR-20a-5p hsa-miR-25-3p hsa-miR-92a-3p
4	SF3B3	hsa-miR-106a-5p hsa-miR-15b-5p hsa-miR-16-5p hsa-miR-34a-5p
4	SGTB	hsa-miR-106a-5p hsa-miR-17-5p hsa-miR-20a-5p hsa-miR-20b-5p
4	SKIL	hsa-miR-106a-5p hsa-miR-17-5p hsa-miR-20a-5p hsa-miR-20b-5p
4	SOX4	hsa-miR-106a-5p hsa-miR-20a-5p hsa-miR-25-3p hsa-miR-92a-3p
4	SPRED1	hsa-miR-126-3p hsa-miR-15a-5p hsa-miR-15b-5p hsa-miR-16-5p
4	SQSTM1	hsa-miR-16-5p hsa-miR-17-5p hsa-miR-20a-5p hsa-miR-20b-5p
4	SRPRB	hsa-miR-15a-5p hsa-miR-15b-5p hsa-miR-16-5p hsa-miR-92a-3p
4	TMEM245	hsa-miR-15a-5p hsa-miR-15b-5p hsa-miR-16-5p hsa-miR-93-3p
4	TTC9	hsa-miR-106a-5p hsa-miR-17-5p hsa-miR-20a-5p hsa-miR-20b-5p
4	UBE2Q1	hsa-miR-15a-5p hsa-miR-15b-5p hsa-miR-16-5p hsa-miR-197-5p
4	USP31	hsa-miR-15a-5p hsa-miR-15b-5p hsa-miR-16-5p hsa-miR-92a-3p
4	YTHDC1	hsa-miR-106a-5p hsa-miR-15a-5p hsa-miR-15b-5p hsa-miR-16-5p
4	ZBTB7A	hsa-miR-106a-5p hsa-miR-17-5p hsa-miR-20a-5p hsa-miR-20b-5p
4	ZFHX4	hsa-miR-15a-5p hsa-miR-15b-5p hsa-miR-16-5p hsa-miR-92a-3p
4	ZNF264	hsa-miR-106a-5p hsa-miR-20a-5p hsa-miR-25-3p hsa-miR-92a-3p
3	ABCF2	hsa-miR-16-5p hsa-miR-25-3p hsa-miR-92a-3p
3	ACOX1	hsa-miR-15a-5p hsa-miR-15b-5p hsa-miR-16-5p
3	ACVR1B	hsa-miR-15a-5p hsa-miR-197-3p hsa-miR-20a-5p
3	ACVR2A	hsa-miR-15a-5p hsa-miR-15b-5p hsa-miR-16-5p
3	AGFG2	hsa-miR-17-5p hsa-miR-20a-5p hsa-miR-20b-5p
3	AMOTL1	hsa-miR-15a-5p hsa-miR-15b-5p hsa-miR-16-5p
3	ANAPC13	hsa-miR-15a-5p hsa-miR-15b-5p hsa-miR-16-5p

3	ANKIB1	hsa-miR-20a-5p hsa-miR-25-3p hsa-miR-92a-3p
3	ANKRD52	hsa-miR-106a-5p hsa-miR-20a-5p hsa-miR-629-3p
3	ANP32E	hsa-miR-25-3p hsa-miR-877-3p hsa-miR-92a-3p
3	ARAP2	hsa-miR-17-5p hsa-miR-20a-5p hsa-miR-20b-5p
3	ARFGEF2	hsa-miR-20a-5p hsa-miR-766-3p hsa-miR-92a-3p
3	ARHGAP1	hsa-miR-20a-5p hsa-miR-25-5p hsa-miR-34a-5p
3	ARHGAP35	hsa-miR-17-5p hsa-miR-20a-5p hsa-miR-20b-5p
3	ARHGDIA	hsa-miR-15a-5p hsa-miR-15b-5p hsa-miR-16-5p
3	ATP5G3	hsa-miR-15a-5p hsa-miR-15b-5p hsa-miR-16-5p
3	AXIN2	hsa-miR-15a-5p hsa-miR-15b-5p hsa-miR-16-5p
3	B3GNT2	hsa-miR-15a-5p hsa-miR-15b-5p hsa-miR-16-5p
3	B4GALT1	hsa-miR-15a-5p hsa-miR-15b-5p hsa-miR-16-5p
3	BAG4	hsa-miR-15a-5p hsa-miR-15b-5p hsa-miR-16-5p
3	BCL7A	hsa-miR-15a-5p hsa-miR-15b-5p hsa-miR-16-5p
3	CA8	hsa-miR-15a-5p hsa-miR-15b-5p hsa-miR-16-5p
3	CACUL1	hsa-miR-15a-5p hsa-miR-15b-5p hsa-miR-16-5p
3	CAMK2N2	hsa-miR-17-5p hsa-miR-20a-5p hsa-miR-20b-5p
3	CAMTA1	hsa-miR-17-5p hsa-miR-20a-5p hsa-miR-20b-5p
3	CARD10	hsa-miR-15a-5p hsa-miR-15b-5p hsa-miR-16-5p
3	CBX2	hsa-miR-15a-5p hsa-miR-15b-5p hsa-miR-16-5p
3	CBX4	hsa-miR-15a-5p hsa-miR-15b-5p hsa-miR-16-5p
3	CBX5	hsa-miR-17-5p hsa-miR-20a-5p hsa-miR-92a-3p
3	CBX6	hsa-miR-15a-5p hsa-miR-15b-5p hsa-miR-16-5p
3	CCNE1	hsa-miR-15a-5p hsa-miR-15b-5p hsa-miR-16-5p
3	CCNT2	hsa-miR-15a-5p hsa-miR-15b-5p hsa-miR-16-5p
3	CDC37L1	hsa-miR-15a-5p hsa-miR-15b-5p hsa-miR-16-5p
3	CDC42SE2	hsa-miR-15a-5p hsa-miR-15b-5p hsa-miR-16-5p
3	CDCA4	hsa-miR-15a-5p hsa-miR-15b-5p hsa-miR-16-5p
3	CDK17	hsa-miR-15a-5p hsa-miR-15b-5p hsa-miR-16-5p
3	CDS2	hsa-miR-15a-5p hsa-miR-15b-5p hsa-miR-16-5p
3	CDV3	hsa-miR-15a-5p hsa-miR-15b-5p hsa-miR-16-5p
3	CEP104	hsa-miR-106a-5p hsa-miR-17-5p hsa-miR-20a-5p
3	CEP55	hsa-miR-15a-5p hsa-miR-15b-5p hsa-miR-16-5p
3	CHAC1	hsa-miR-15a-5p hsa-miR-15b-5p hsa-miR-16-5p
3	CHMP3	hsa-miR-15a-5p hsa-miR-15b-5p hsa-miR-16-5p
3	CLIC4	hsa-miR-106a-5p hsa-miR-17-5p hsa-miR-20a-5p
3	CMPK1	hsa-miR-16-5p hsa-miR-17-5p hsa-miR-20b-5p
3	CMTM4	hsa-miR-15a-5p hsa-miR-15b-5p hsa-miR-16-5p
3	CNOT4	hsa-miR-106a-5p hsa-miR-20a-5p hsa-miR-34a-5p
3	CREG1	hsa-miR-15a-5p hsa-miR-15b-5p hsa-miR-16-5p
3	CRIM1	hsa-miR-15b-5p hsa-miR-16-5p hsa-miR-20a-5p
3	CSDE1	hsa-miR-15a-5p hsa-miR-15b-5p hsa-miR-16-5p
3	CYP26B1	hsa-miR-15a-5p hsa-miR-15b-5p hsa-miR-16-5p
3	DDIT4	hsa-miR-181a-5p hsa-miR-221-3p hsa-miR-92a-3p
3	DNAJA1	hsa-miR-15a-5p hsa-miR-15b-5p hsa-miR-16-5p
3	DUSP18	hsa-miR-17-5p hsa-miR-20a-5p hsa-miR-20b-5p
3	DYNC1LI2	hsa-miR-106a-5p hsa-miR-181a-5p hsa-miR-20a-5p
3	EDC3	hsa-miR-15a-5p hsa-miR-15b-5p hsa-miR-16-5p
3	FASN	hsa-miR-15a-5p hsa-miR-15b-5p hsa-miR-16-5p
3	FBXO3	hsa-miR-15a-5p hsa-miR-16-5p hsa-miR-20a-5p
3	FCF1	hsa-miR-15a-5p hsa-miR-15b-5p hsa-miR-16-5p
3	FCHO2	hsa-miR-17-5p hsa-miR-20a-5p hsa-miR-20b-5p

3	FICD	hsa-miR-17-5p hsa-miR-20a-5p hsa-miR-20b-5p
3	FNDC3B	hsa-miR-16-5p hsa-miR-25-3p hsa-miR-92a-3p
3	GALNT1	hsa-miR-15a-5p hsa-miR-15b-5p hsa-miR-16-5p
3	GATAD2B	hsa-miR-221-5p hsa-miR-25-3p hsa-miR-92a-3p
3	GFPT1	hsa-miR-16-5p hsa-miR-34a-5p hsa-miR-34b-3p
3	GOSR1	hsa-miR-15a-5p hsa-miR-15b-5p hsa-miR-16-5p
3	GRB2	hsa-miR-15a-5p hsa-miR-15b-5p hsa-miR-16-5p
3	GSK3B	hsa-let-7b-3p hsa-miR-877-3p hsa-miR-92a-3p
3	HIPK1	hsa-miR-7-1-3p hsa-miR-766-5p hsa-miR-92a-3p
3	HNF4A	hsa-miR-197-3p hsa-miR-34a-5p hsa-miR-766-3p
3	HNRNPA2B1	hsa-miR-15a-5p hsa-miR-15b-5p hsa-miR-16-5p
3	IGF1R	hsa-miR-16-5p hsa-miR-34a-3p hsa-miR-885-5p
3	IGFBP5	hsa-miR-193a-3p hsa-miR-197-3p hsa-miR-411-3p
3	IPPK	hsa-miR-15a-5p hsa-miR-15b-5p hsa-miR-16-5p
3	IRGQ	hsa-miR-34a-5p hsa-miR-766-3p hsa-miR-92a-3p
3	ITPKB	hsa-miR-17-5p hsa-miR-20a-5p hsa-miR-20b-5p
3	KMT2D	hsa-miR-15b-5p hsa-miR-34a-5p hsa-miR-766-3p
3	LAMP2	hsa-miR-15a-5p hsa-miR-15b-5p hsa-miR-16-5p
3	LAMTOR1	hsa-miR-106a-5p hsa-miR-16-5p hsa-miR-20a-5p
3	LDLR	hsa-miR-106a-5p hsa-miR-20a-5p hsa-miR-7-1-3p
3	LHFPL2	hsa-miR-221-3p hsa-miR-25-3p hsa-miR-92a-3p
3	LRIG2	hsa-miR-15a-5p hsa-miR-15b-5p hsa-miR-16-5p
3	LRPPRC	hsa-miR-15a-5p hsa-miR-15b-5p hsa-miR-16-5p
3	LSM11	hsa-miR-15a-5p hsa-miR-15b-5p hsa-miR-16-5p
3	MAFK	hsa-miR-15a-5p hsa-miR-15b-5p hsa-miR-16-5p
3	MAPK1	hsa-miR-106a-5p hsa-miR-20a-5p hsa-miR-766-3p
3	MIB1	hsa-miR-15a-5p hsa-miR-15b-5p hsa-miR-16-5p
3	MOAP1	hsa-miR-25-3p hsa-miR-34a-5p hsa-miR-92a-3p
3	MTHFR	hsa-miR-15a-5p hsa-miR-15b-5p hsa-miR-16-5p
3	MTMR3	hsa-miR-15a-5p hsa-miR-15b-5p hsa-miR-16-5p
3	N4BP1	hsa-miR-15a-5p hsa-miR-15b-5p hsa-miR-16-5p
3	NAA25	hsa-miR-15a-5p hsa-miR-15b-5p hsa-miR-16-5p
3	NAPG	hsa-miR-15a-5p hsa-miR-15b-5p hsa-miR-16-5p
3	NCOR2	hsa-miR-15a-5p hsa-miR-15b-5p hsa-miR-16-5p
3	NETO2	hsa-miR-106a-5p hsa-miR-17-5p hsa-miR-20a-5p
3	NRAS	hsa-miR-20a-5p hsa-miR-25-3p hsa-miR-92a-3p
3	NRBP1	hsa-miR-106a-5p hsa-miR-20a-5p hsa-miR-766-5p
3	NUP160	hsa-miR-15b-5p hsa-miR-16-5p hsa-miR-93-3p
3	OGT	hsa-miR-15a-5p hsa-miR-15b-5p hsa-miR-16-5p
3	PAG1	hsa-miR-15a-5p hsa-miR-15b-5p hsa-miR-16-5p
3	PAK2	hsa-miR-15a-5p hsa-miR-15b-5p hsa-miR-16-5p
3	PAPD7	hsa-miR-16-2-3p hsa-miR-25-3p hsa-miR-92a-3p
3	PDE4D	hsa-miR-15a-5p hsa-miR-15b-5p hsa-miR-16-5p
3	PEG10	hsa-miR-32-3p hsa-miR-34a-5p hsa-miR-766-5p
3	PGD	hsa-miR-15a-5p hsa-miR-15b-5p hsa-miR-16-5p
3	PHTF2	hsa-miR-106a-5p hsa-miR-20a-5p hsa-miR-92a-3p
3	PI4K2B	hsa-miR-15a-5p hsa-miR-15b-5p hsa-miR-16-5p
3	PISD	hsa-miR-15a-5p hsa-miR-15b-5p hsa-miR-16-5p
3	PLAG1	hsa-miR-15a-5p hsa-miR-15b-5p hsa-miR-16-5p
3	PPAP2B	hsa-miR-15a-5p hsa-miR-15b-5p hsa-miR-16-5p
3	PPIG	hsa-miR-15a-5p hsa-miR-15b-5p hsa-miR-16-5p
3	PPIL1	hsa-miR-15a-5p hsa-miR-15b-5p hsa-miR-16-5p

3	PPP1R15B	hsa-miR-106a-5p hsa-miR-20a-5p hsa-miR-221-3p
3	PRDM4	hsa-miR-15a-5p hsa-miR-15b-5p hsa-miR-16-5p
3	PRNP	hsa-miR-17-5p hsa-miR-20b-5p hsa-miR-221-5p
3	PSAT1	hsa-miR-15a-5p hsa-miR-15b-5p hsa-miR-16-5p
3	PSKH1	hsa-miR-15a-5p hsa-miR-15b-5p hsa-miR-16-5p
3	PTPDC1	hsa-miR-16-5p hsa-miR-17-5p hsa-miR-20a-5p
3	RACGAP1	hsa-miR-15a-5p hsa-miR-15b-5p hsa-miR-16-5p
3	RAD23B	hsa-miR-15a-5p hsa-miR-15b-5p hsa-miR-16-5p
3	RAPH1	hsa-miR-15a-5p hsa-miR-15b-5p hsa-miR-16-5p
3	RBM27	hsa-miR-15b-5p hsa-miR-25-3p hsa-miR-92a-3p
3	RECK	hsa-miR-15a-5p hsa-miR-15b-5p hsa-miR-16-5p
3	REEP3	hsa-miR-106a-5p hsa-miR-20a-5p hsa-miR-34a-5p
3	RIF1	hsa-miR-15a-5p hsa-miR-15b-5p hsa-miR-16-5p
3	RLIM	hsa-miR-106a-5p hsa-miR-15b-5p hsa-miR-20a-5p
3	RNF138	hsa-miR-15a-5p hsa-miR-15b-5p hsa-miR-16-5p
3	RNF149	hsa-miR-15a-5p hsa-miR-15b-5p hsa-miR-16-5p
3	RNF168	hsa-miR-15a-5p hsa-miR-15b-5p hsa-miR-16-5p
3	RNF44	hsa-miR-221-3p hsa-miR-25-3p hsa-miR-92a-3p
3	RNMT	hsa-miR-15a-5p hsa-miR-15b-5p hsa-miR-16-5p
3	RPS6KA3	hsa-miR-15a-5p hsa-miR-15b-5p hsa-miR-16-5p
3	RRAGD	hsa-miR-106a-5p hsa-miR-20a-5p hsa-miR-221-5p
3	RRN3	hsa-miR-20a-5p hsa-miR-25-3p hsa-miR-92a-3p
3	RTN4	hsa-miR-15a-5p hsa-miR-15b-5p hsa-miR-16-5p
3	SAMD12	hsa-miR-17-5p hsa-miR-20a-5p hsa-miR-20b-5p
3	SAMD9L	hsa-miR-17-5p hsa-miR-20a-5p hsa-miR-20b-5p
3	SCAMP4	hsa-miR-15a-5p hsa-miR-15b-5p hsa-miR-16-5p
3	SESN2	hsa-miR-17-3p hsa-miR-20a-5p hsa-miR-34a-5p
3	SESTD1	hsa-miR-15a-5p hsa-miR-15b-5p hsa-miR-16-5p
3	SHOC2	hsa-miR-106a-5p hsa-miR-16-5p hsa-miR-34a-5p
3	SLC22A23	hsa-miR-17-5p hsa-miR-20a-5p hsa-miR-20b-5p
3	SLC25A22	hsa-miR-15a-5p hsa-miR-15b-5p hsa-miR-16-5p
3	SLC25A36	hsa-miR-221-3p hsa-miR-25-3p hsa-miR-92a-3p
3	SLC2A3	hsa-miR-15a-5p hsa-miR-15b-5p hsa-miR-16-5p
3	SLC7A11	hsa-miR-181a-5p hsa-miR-20a-5p hsa-miR-92a-3p
3	SMAD5	hsa-miR-17-5p hsa-miR-20a-5p hsa-miR-20b-5p
3	SNTB2	hsa-miR-15a-5p hsa-miR-15b-5p hsa-miR-16-5p
3	SREK1	hsa-miR-15a-5p hsa-miR-15b-5p hsa-miR-16-5p
3	STK38	hsa-miR-15a-5p hsa-miR-15b-5p hsa-miR-16-5p
3	STXBP3	hsa-miR-15a-5p hsa-miR-15b-5p hsa-miR-16-5p
3	TADA2B	hsa-miR-15a-5p hsa-miR-15b-5p hsa-miR-16-5p
3	TAOK1	hsa-miR-15a-5p hsa-miR-15b-5p hsa-miR-16-5p
3	TBC1D20	hsa-miR-15a-5p hsa-miR-15b-5p hsa-miR-16-5p
3	TBCCD1	hsa-miR-15a-5p hsa-miR-15b-5p hsa-miR-16-5p
3	TBL1XR1	hsa-miR-15a-5p hsa-miR-15b-5p hsa-miR-16-5p
3	TBPL1	hsa-miR-15a-5p hsa-miR-15b-5p hsa-miR-16-5p
3	TCF3	hsa-miR-15a-5p hsa-miR-15b-5p hsa-miR-16-5p
3	TIMM13	hsa-miR-15a-5p hsa-miR-15b-5p hsa-miR-16-5p
3	TM4SF1	hsa-miR-15a-5p hsa-miR-15b-5p hsa-miR-16-5p
3	TM9SF2	hsa-miR-15a-5p hsa-miR-15b-5p hsa-miR-16-5p
3	TM9SF3	hsa-miR-15b-5p hsa-miR-16-2-3p hsa-miR-34a-5p
3	TMEM109	hsa-miR-15a-5p hsa-miR-16-5p hsa-miR-34a-5p
3	TMEM167A	hsa-miR-17-5p hsa-miR-20a-5p hsa-miR-20b-5p

3	MEM189-UBE2	hsa-miR-15a-5p hsa-miR-15b-5p hsa-miR-16-5p
3	TMEM55B	hsa-miR-15a-5p hsa-miR-15b-5p hsa-miR-16-5p
3	TNKS2	hsa-miR-106a-5p hsa-miR-20a-5p hsa-miR-221-3p
3	TNPO1	hsa-miR-16-2-3p hsa-miR-16-5p hsa-miR-92a-3p
3	TRAM2	hsa-miR-25-3p hsa-miR-34a-3p hsa-miR-92a-3p
3	TRIM35	hsa-miR-15a-5p hsa-miR-15b-5p hsa-miR-16-5p
3	TSPAN3	hsa-miR-15b-5p hsa-miR-16-5p hsa-miR-197-3p
3	TTC1	hsa-miR-15a-5p hsa-miR-15b-5p hsa-miR-16-5p
3	TUBB	hsa-miR-15a-5p hsa-miR-15b-5p hsa-miR-16-5p
3	TXLNA	hsa-miR-15b-5p hsa-miR-877-3p hsa-miR-92a-3p
3	UBE2Q2	hsa-miR-17-5p hsa-miR-20a-5p hsa-miR-20b-5p
3	UBE2Z	hsa-miR-16-5p hsa-miR-25-3p hsa-miR-92a-3p
3	UBN2	hsa-miR-15a-5p hsa-miR-15b-5p hsa-miR-16-5p
3	WDR37	hsa-miR-20a-5p hsa-miR-32-3p hsa-miR-92a-3p
3	WIPF2	hsa-miR-106a-5p hsa-miR-20a-5p hsa-miR-92a-3p
3	WIPI2	hsa-miR-15a-5p hsa-miR-15b-5p hsa-miR-16-5p
3	XIAP	hsa-miR-20a-5p hsa-miR-30b-3p hsa-miR-34b-5p
3	YOD1	hsa-miR-106a-5p hsa-miR-20a-5p hsa-miR-550a-3p
3	YRDC	hsa-miR-15a-5p hsa-miR-15b-5p hsa-miR-16-5p
3	ZBTB10	hsa-miR-15a-5p hsa-miR-15b-5p hsa-miR-16-5p
3	ZBTB18	hsa-miR-106a-5p hsa-miR-15a-3p hsa-miR-20a-5p
3	ZBTB34	hsa-miR-15a-5p hsa-miR-15b-5p hsa-miR-16-5p
3	ZFYVE21	hsa-miR-20a-5p hsa-miR-25-3p hsa-miR-92a-3p
3	ZNF267	hsa-miR-15a-5p hsa-miR-16-5p hsa-miR-92a-3p
3	ZNF367	hsa-miR-15a-5p hsa-miR-15b-5p hsa-miR-16-5p
3	ZNF622	hsa-miR-15a-5p hsa-miR-15b-5p hsa-miR-16-5p
3	ZNF704	hsa-miR-15a-5p hsa-miR-15b-5p hsa-miR-16-5p
3	ZNF770	hsa-miR-17-5p hsa-miR-20a-5p hsa-miR-20b-5p
3	ZNRF2	hsa-miR-15a-5p hsa-miR-15b-5p hsa-miR-16-5p
3	ZNRF3	hsa-miR-15a-5p hsa-miR-15b-5p hsa-miR-16-5p
2	ABHD2	hsa-miR-16-5p hsa-miR-92a-3p
2	ABI2	hsa-miR-136-5p hsa-miR-193a-3p
2	ACAP2	hsa-miR-20a-5p hsa-miR-766-3p
2	ADSS	hsa-miR-16-5p hsa-miR-20a-3p
2	AGPAT5	hsa-miR-15b-5p hsa-miR-16-5p
2	ANKRD40	hsa-miR-30b-3p hsa-miR-766-5p
2	ANKRD50	hsa-miR-106a-5p hsa-miR-20a-5p
2	AP3D1	hsa-miR-16-5p hsa-miR-20a-5p
2	ARF1	hsa-miR-25-3p hsa-miR-92a-3p
2	ARL6IP1	hsa-miR-16-2-3p hsa-miR-543
2	ARNTL2	hsa-miR-25-3p hsa-miR-92a-3p
2	ARPP19	hsa-miR-7-1-3p hsa-miR-92a-3p
2	ATAD5	hsa-miR-15a-5p hsa-miR-16-5p
2	ATG14	hsa-miR-15a-5p hsa-miR-16-5p
2	ATP6V0E1	hsa-miR-15b-5p hsa-miR-16-5p
2	AVL9	hsa-miR-15a-5p hsa-miR-16-5p
2	B2M	hsa-miR-16-2-3p hsa-miR-20a-5p
2	BBX	hsa-miR-106a-5p hsa-miR-20a-5p
2	BCAT1	hsa-miR-25-3p hsa-miR-92a-3p
2	BRI3BP	hsa-miR-106a-5p hsa-miR-20a-5p
2	BRMS1L	hsa-miR-20a-5p hsa-miR-92a-3p
2	BRWD1	hsa-miR-15a-5p hsa-miR-221-3p

2	BTAF1	hsa-miR-15b-5p hsa-miR-16-5p
2	BTG1	hsa-let-7b-3p hsa-miR-498
2	C11orf24	hsa-miR-25-3p hsa-miR-92a-3p
2	C16orf72	hsa-miR-16-5p hsa-miR-377-3p
2	C9orf40	hsa-miR-106a-5p hsa-miR-20a-5p
2	CAV1	hsa-miR-20a-5p hsa-miR-34b-5p
2	CCDC137	hsa-miR-106a-5p hsa-miR-20a-5p
2	CCDC6	hsa-miR-181a-5p hsa-miR-20a-5p
2	CCND3	hsa-miR-16-5p hsa-miR-34a-5p
2	CDC23	hsa-miR-16-5p hsa-miR-34a-5p
2	CDC5L	hsa-miR-16-5p hsa-miR-7-1-3p
2	CDK5R1	hsa-miR-25-3p hsa-miR-92a-3p
2	CDKN1B	hsa-miR-181a-5p hsa-miR-221-3p
2	CENPQ	hsa-miR-106a-5p hsa-miR-20a-5p
2	CEP97	hsa-miR-106a-5p hsa-miR-20a-5p
2	CHUK	hsa-miR-15a-5p hsa-miR-16-5p
2	CKAP2	hsa-miR-106a-5p hsa-miR-20a-5p
2	CNBP	hsa-miR-197-3p hsa-miR-877-3p
2	CNIH1	hsa-miR-25-3p hsa-miR-92a-3p
2	CNNM4	hsa-miR-25-3p hsa-miR-92a-3p
2	CNOT7	hsa-miR-17-5p hsa-miR-20a-5p
2	COL4A1	hsa-miR-16-5p hsa-miR-92a-3p
2	CPEB4	hsa-miR-25-3p hsa-miR-92a-3p
2	CPSF2	hsa-miR-149-3p hsa-miR-193a-3p
2	CPSF7	hsa-miR-15a-5p hsa-miR-15b-5p
2	CREBL2	hsa-miR-15a-5p hsa-miR-16-5p
2	CSNK1A1	hsa-miR-106a-5p hsa-miR-20a-5p
2	CUL5	hsa-miR-181a-5p hsa-miR-92a-3p
2	DCAF17	hsa-miR-15a-5p hsa-miR-15b-5p
2	DCAF8	hsa-miR-106a-5p hsa-miR-20a-5p
2	DCBLD2	hsa-miR-106a-5p hsa-miR-17-5p
2	DDI2	hsa-miR-25-3p hsa-miR-92a-3p
2	DDX3Y	hsa-miR-16-5p hsa-miR-221-3p
2	DLC1	hsa-miR-16-5p hsa-miR-20a-5p
2	DNAJC27	hsa-miR-20a-5p hsa-miR-92a-3p
2	DUSP2	hsa-miR-106a-5p hsa-miR-20a-5p
2	DUSP5	hsa-miR-25-3p hsa-miR-92a-3p
2	DYRK2	hsa-miR-193a-3p hsa-miR-20a-5p
2	E2F1	hsa-miR-149-3p hsa-miR-20a-5p
2	EDEM1	hsa-miR-25-3p hsa-miR-92a-3p
2	EID2B	hsa-miR-25-3p hsa-miR-92a-3p
2	EIF1	hsa-miR-25-3p hsa-miR-92a-3p
2	EIF5B	hsa-miR-15b-5p hsa-miR-16-5p
2	ELAC2	hsa-miR-16-5p hsa-miR-92a-3p
2	EP300	hsa-miR-25-3p hsa-miR-92a-3p
2	EPAS1	hsa-miR-17-5p hsa-miR-20a-5p
2	EPHA4	hsa-miR-106a-5p hsa-miR-20a-5p
2	ERAP1	hsa-miR-20a-5p hsa-miR-92a-3p
2	ERGIC2	hsa-miR-25-3p hsa-miR-92a-3p
2	ERLIN2	hsa-miR-16-5p hsa-miR-34a-5p
2	ETF1	hsa-miR-17-5p hsa-miR-20b-5p
2	ETV1	hsa-miR-17-5p hsa-miR-20a-5p

2	F2R	hsa-miR-20a-5p hsa-miR-20b-5p
2	F3	hsa-miR-106a-5p hsa-miR-20a-5p
2	FAF2	hsa-miR-17-5p hsa-miR-20a-5p
2	FAM160B1	hsa-miR-106a-5p hsa-miR-20a-5p
2	FBXW7	hsa-miR-16-5p hsa-miR-25-3p
2	FJX1	hsa-miR-106a-5p hsa-miR-20a-5p
2	FKBP14	hsa-miR-106a-5p hsa-miR-20a-5p
2	FOS	hsa-miR-221-3p hsa-miR-543
2	FOXC1	hsa-miR-17-5p hsa-miR-20a-5p
2	FOXJ2	hsa-miR-106a-5p hsa-miR-20a-5p
2	FOXJ3	hsa-miR-106a-5p hsa-miR-20a-5p
2	FOXN2	hsa-miR-25-3p hsa-miR-92a-3p
2	FRS2	hsa-miR-106a-5p hsa-miR-20a-5p
2	G3BP2	hsa-miR-16-5p hsa-miR-92a-3p
2	GAB1	hsa-miR-17-5p hsa-miR-20a-5p
2	GDF11	hsa-miR-106a-5p hsa-miR-20a-5p
2	GDI2	hsa-miR-15a-5p hsa-miR-20a-5p
2	GEMIN5	hsa-miR-34a-5p hsa-miR-877-3p
2	GFPT2	hsa-miR-25-3p hsa-miR-92a-3p
2	GID4	hsa-miR-25-3p hsa-miR-92a-3p
2	GLO1	hsa-miR-106a-5p hsa-miR-20a-5p
2	GNAQ	hsa-miR-25-3p hsa-miR-92a-3p
2	GOLGA1	hsa-miR-17-5p hsa-miR-20a-5p
2	GOLGA3	hsa-miR-25-3p hsa-miR-92a-3p
2	GOLGA8A	hsa-miR-25-3p hsa-miR-92a-3p
2	GOLGA8B	hsa-miR-25-3p hsa-miR-92a-3p
2	GPAM	hsa-miR-16-5p hsa-miR-20a-5p
2	GPR137B	hsa-miR-106a-5p hsa-miR-20a-5p
2	GTF2A1	hsa-miR-25-3p hsa-miR-92a-3p
2	GTF2H1	hsa-miR-15a-5p hsa-miR-16-5p
2	H3F3B	hsa-miR-25-3p hsa-miR-92a-3p
2	HAS2	hsa-miR-106a-5p hsa-miR-20a-5p
2	HIPK3	hsa-miR-106a-5p hsa-miR-92a-3p
2	HMGB3	hsa-miR-17-5p hsa-miR-20b-5p
2	HNRNPA0	hsa-miR-221-3p hsa-miR-376a-3p
2	HSDL2	hsa-miR-15a-5p hsa-miR-16-5p
2	HSPA8	hsa-miR-15b-5p hsa-miR-16-5p
2	ICAM1	hsa-miR-17-3p hsa-miR-221-3p
2	INSIG1	hsa-miR-25-3p hsa-miR-92a-3p
2	KDM6B	hsa-miR-106a-5p hsa-miR-20a-5p
2	KDSR	hsa-miR-16-5p hsa-miR-92a-3p
2	KHDRBS1	hsa-miR-877-3p hsa-miR-92a-3p
2	KIAA1191	hsa-miR-106a-5p hsa-miR-20a-5p
2	KIT	hsa-miR-221-3p hsa-miR-34a-5p
2	KLF3	hsa-miR-106a-5p hsa-miR-20a-5p
2	KLF4	hsa-miR-25-3p hsa-miR-34a-5p
2	KLHDC10	hsa-miR-25-3p hsa-miR-92a-3p
2	KLHL28	hsa-miR-106a-5p hsa-miR-20a-5p
2	KMT2A	hsa-miR-16-5p hsa-miR-193a-3p
2	LAMC1	hsa-miR-16-5p hsa-miR-20a-5p
2	LARP1	hsa-miR-16-5p hsa-miR-34a-5p
2	LASP1	hsa-miR-106a-5p hsa-miR-20a-5p

2	LPGAT1	hsa-miR-181a-5p hsa-miR-20a-5p
2	LRPAP1	hsa-miR-106a-5p hsa-miR-20a-5p
2	LZIC	hsa-miR-106a-5p hsa-miR-20a-5p
2	MAP1B	hsa-miR-25-3p hsa-miR-411-5p
2	MAP2K1	hsa-miR-181a-5p hsa-miR-34a-5p
2	MAP2K4	hsa-miR-25-3p hsa-miR-92a-3p
2	MAP3K2	hsa-miR-106a-5p hsa-miR-20a-5p
2	MAPRE3	hsa-miR-17-5p hsa-miR-20a-5p
2	MAZ	hsa-miR-149-3p hsa-miR-30b-3p
2	MCL1	hsa-miR-193a-3p hsa-miR-20a-5p
2	MED19	hsa-miR-25-3p hsa-miR-92a-3p
2	MEF2D	hsa-miR-654-5p hsa-miR-92a-3p
2	METAP1	hsa-miR-181a-5p hsa-miR-34a-5p
2	MFN1	hsa-miR-106a-5p hsa-miR-20a-5p
2	MKNK2	hsa-miR-17-5p hsa-miR-20a-5p
2	MSMO1	hsa-miR-106a-5p hsa-miR-20a-5p
2	MTF1	hsa-miR-106a-5p hsa-miR-20a-5p
2	MYBL1	hsa-miR-15a-5p hsa-miR-221-3p
2	NAA50	hsa-miR-20a-5p hsa-miR-766-5p
2	NABP1	hsa-miR-106a-5p hsa-miR-20a-5p
2	NCL	hsa-miR-197-3p hsa-miR-877-3p
2	NFATC2IP	hsa-miR-20a-5p hsa-miR-92a-3p
2	NUS1	hsa-miR-197-3p hsa-miR-877-3p
2	ORAI2	hsa-miR-1224-3p hsa-miR-16-5p
2	PANK3	hsa-miR-20a-5p hsa-miR-221-3p
2	PAWR	hsa-miR-25-3p hsa-miR-92a-3p
2	PDHX	hsa-miR-15a-5p hsa-miR-16-5p
2	PDZD8	hsa-miR-25-3p hsa-miR-92a-3p
2	PFKP	hsa-miR-106a-5p hsa-miR-20a-5p
2	PGM2L1	hsa-miR-17-5p hsa-miR-20a-5p
2	PIP4K2C	hsa-miR-20a-5p hsa-miR-877-3p
2	PIP5K1C	hsa-miR-25-3p hsa-miR-92a-3p
2	PNRC2	hsa-miR-181a-5p hsa-miR-374b-3p
2	POLR3A	hsa-miR-106a-5p hsa-miR-16-5p
2	PPIF	hsa-miR-16-5p hsa-miR-766-5p
2	PPP1R37	hsa-miR-25-3p hsa-miR-92a-3p
2	PPP3R1	hsa-miR-20a-5p hsa-miR-34a-5p
2	PPT1	hsa-miR-15b-5p hsa-miR-16-5p
2	PRKCD	hsa-miR-15a-5p hsa-miR-16-5p
2	PTCD3	hsa-miR-16-5p hsa-miR-34a-5p
2	PTGES2	hsa-miR-25-3p hsa-miR-92a-3p
2	PTGFRN	hsa-miR-106a-5p hsa-miR-20a-5p
2	PTGS2	hsa-miR-16-5p hsa-miR-181a-5p
2	PTPN4	hsa-miR-106a-5p hsa-miR-20a-5p
2	PURB	hsa-miR-106a-5p hsa-miR-20a-5p
2	QSER1	hsa-miR-7-1-3p hsa-miR-92a-3p
2	RAB12	hsa-miR-16-5p hsa-miR-20a-5p
2	RAB15	hsa-miR-15a-5p hsa-miR-16-5p
2	RAB22A	hsa-miR-106a-5p hsa-miR-20a-5p
2	RAB9B	hsa-miR-15a-5p hsa-miR-16-5p
2	RAC1	hsa-miR-136-5p hsa-miR-574-3p
2	RAN	hsa-miR-106a-5p hsa-miR-20a-5p

2	RB1	hsa-miR-106a-5p hsa-miR-20a-5p
2	RBFOX2	hsa-miR-432-3p hsa-miR-92a-3p
2	RBL2	hsa-miR-106a-5p hsa-miR-20a-5p
2	RBM15B	hsa-miR-34a-5p hsa-miR-93-3p
2	RBM33	hsa-miR-20a-3p hsa-miR-34a-5p
2	REST	hsa-miR-106a-5p hsa-miR-20a-5p
2	RPL27A	hsa-miR-15b-5p hsa-miR-16-5p
2	RPS4X	hsa-miR-154-5p hsa-miR-16-2-3p
2	RSBN1	hsa-miR-25-3p hsa-miR-92a-3p
2	RUFY2	hsa-miR-106a-5p hsa-miR-20a-5p
2	RUNX3	hsa-miR-106a-5p hsa-miR-20a-5p
2	SAR1A	hsa-miR-34a-5p hsa-miR-34b-3p
2	SBNO1	hsa-miR-15a-5p hsa-miR-19b-1-5p
2	SCAMP2	hsa-miR-106a-5p hsa-miR-20a-5p
2	SEMA7A	hsa-miR-106a-5p hsa-miR-20a-5p
2	SERTAD3	hsa-miR-25-3p hsa-miR-92a-3p
2	SETD1B	hsa-miR-16-5p hsa-miR-92a-3p
2	SGPP1	hsa-miR-34a-5p hsa-miR-92a-3p
2	SLC1A5	hsa-miR-15b-5p hsa-miR-16-5p
2	SLC25A25	hsa-miR-181a-5p hsa-miR-877-3p
2	SLC30A1	hsa-miR-106a-5p hsa-miR-20a-5p
2	SLC35F5	hsa-miR-106a-5p hsa-miR-20a-5p
2	SLC5A3	hsa-miR-20a-5p hsa-miR-877-3p
2	SLC6A8	hsa-miR-877-3p hsa-miR-92a-3p
2	SLX4	hsa-miR-25-3p hsa-miR-92a-3p
2	SMDT1	hsa-miR-15a-5p hsa-miR-15b-5p
2	SMOC1	hsa-miR-106a-5p hsa-miR-20a-5p
2	SNX6	hsa-miR-15a-5p hsa-miR-16-5p
2	SORT1	hsa-miR-181a-5p hsa-miR-34a-5p
2	SOX5	hsa-miR-15a-5p hsa-miR-16-5p
2	SPCS3	hsa-miR-16-5p hsa-miR-92a-3p
2	SPEN	hsa-miR-16-5p hsa-miR-877-3p
2	SPRYD4	hsa-miR-25-3p hsa-miR-92a-3p
2	SRSF2	hsa-miR-20a-5p hsa-miR-221-3p
2	STAG2	hsa-miR-136-5p hsa-miR-181a-5p
2	SYPL1	hsa-miR-15a-5p hsa-miR-16-5p
2	TEF	hsa-miR-25-3p hsa-miR-92a-3p
2	TET3	hsa-miR-17-5p hsa-miR-20b-5p
2	THBS1	hsa-miR-411-3p hsa-miR-7-1-3p
2	TMEM127	hsa-miR-106a-5p hsa-miR-20a-5p
2	TMEM200C	hsa-miR-106a-5p hsa-miR-20a-5p
2	TMEM43	hsa-miR-15b-5p hsa-miR-16-5p
2	TMEM9B	hsa-miR-106a-5p hsa-miR-20a-5p
2	TMF1	hsa-miR-32-3p hsa-miR-92a-3p
2	TMOD3	hsa-miR-17-5p hsa-miR-20b-5p
2	TNFRSF10B	hsa-miR-106a-5p hsa-miR-20a-5p
2	TNFSF9	hsa-miR-15b-5p hsa-miR-16-5p
2	TOB1	hsa-miR-25-3p hsa-miR-92a-3p
2	TSFM	hsa-miR-25-3p hsa-miR-877-3p
2	TSN	hsa-miR-34a-5p hsa-miR-432-3p
2	TULP4	hsa-miR-25-3p hsa-miR-92a-3p
2	UBE2R2	hsa-miR-92a-3p hsa-miR-93-3p

2	UBE3C	hsa-miR-15b-5p hsa-miR-16-5p
2	UBFD1	hsa-miR-16-5p hsa-miR-20a-5p
2	UGCG	hsa-miR-106a-5p hsa-miR-20a-5p
2	USP15	hsa-miR-15a-5p hsa-miR-16-5p
2	VEGFA	hsa-miR-15a-5p hsa-miR-16-5p
2	VPS4B	hsa-miR-25-3p hsa-miR-92a-3p
2	VPS53	hsa-miR-221-3p hsa-miR-877-3p
2	WASL	hsa-miR-17-5p hsa-miR-92a-3p
2	WDR82	hsa-miR-17-5p hsa-miR-193a-3p
2	WNT3A	hsa-miR-15a-5p hsa-miR-16-5p
2	XRN1	hsa-miR-34a-5p hsa-miR-92a-3p
2	YWHAZ	hsa-miR-17-5p hsa-miR-20b-5p
2	ZBTB4	hsa-miR-17-5p hsa-miR-20a-5p
2	ZDHHC5	hsa-miR-25-3p hsa-miR-92a-3p
2	ZNF202	hsa-miR-106a-5p hsa-miR-20a-5p
2	ZNF24	hsa-miR-25-3p hsa-miR-92a-3p
2	ZNF460	hsa-miR-25-3p hsa-miR-766-3p
2	ZNF703	hsa-miR-550a-3p hsa-miR-92a-3p
2	ZRANB2	hsa-miR-16-5p hsa-miR-877-3p
1	AGO1	hsa-miR-34a-5p
1	AAAS	hsa-miR-16-5p
1	AADAT	hsa-miR-16-5p
1	AAGAB	hsa-miR-93-3p
1	AAK1	hsa-miR-20a-5p
1	ABCA1	hsa-miR-20a-5p
1	ABCA3	hsa-miR-20a-5p
1	ABCB7	hsa-miR-16-5p
1	ABCF3	hsa-miR-766-3p
1	ABHD10	hsa-miR-629-3p
1	ACSL3	hsa-miR-377-5p
1	ACTR1A	hsa-miR-15a-5p
1	ADNP	hsa-miR-34a-5p
1	ADO	hsa-miR-34a-5p
1	AFG3L2	hsa-miR-16-5p
1	AFTPH	hsa-miR-181a-5p
1	AGK	hsa-miR-16-5p
1	AGPAT6	hsa-miR-766-3p
1	AGRN	hsa-miR-16-5p
1	AHCYL1	hsa-miR-16-5p
1	AHCYL2	hsa-miR-16-5p
1	AHR	hsa-miR-377-5p
1	AIM1	hsa-miR-34a-5p
1	AKAP10	hsa-miR-92a-3p
1	AKAP12	hsa-miR-877-3p
1	ALDH18A1	hsa-miR-16-5p
1	ALDH2	hsa-miR-16-5p
1	ALDH9A1	hsa-miR-20a-5p
1	ALG3	hsa-miR-16-5p
1	AMER1	hsa-miR-16-5p
1	AMMECR1L	hsa-miR-15b-5p
1	ANK2	hsa-miR-877-3p
1	ANKRD17	hsa-miR-16-5p

1	ANXA1	hsa-miR-221-3p
1	AP1G1	hsa-miR-20a-5p
1	AP2M1	hsa-miR-16-5p
1	APOL2	hsa-miR-221-3p
1	APP	hsa-miR-16-5p
1	APPL1	hsa-miR-92a-3p
1	ARFIP1	hsa-miR-877-3p
1	ARHGDIB	hsa-miR-34a-5p
1	ARIH1	hsa-miR-16-5p
1	ARL1	hsa-miR-20a-5p
1	ARL10	hsa-miR-16-5p
1	ARL2	hsa-miR-16-5p
1	ARL2BP	hsa-miR-369-3p
1	ARL3	hsa-miR-16-5p
1	ARL5B	hsa-miR-30b-3p
1	ARMCX2	hsa-miR-16-5p
1	ARNT	hsa-miR-221-3p
1	ARRDC3	hsa-miR-877-3p
1	ARSJ	hsa-miR-20a-5p
1	ASB1	hsa-miR-20a-5p
1	ASB6	hsa-miR-16-5p
1	ASNSD1	hsa-miR-15a-5p
1	ASXL3	hsa-miR-221-3p
1	ATF7	hsa-miR-16-5p
1	ATG2B	hsa-miR-20a-5p
1	ATL2	hsa-miR-16-5p
1	ATOX1	hsa-miR-92a-3p
1	ATP6V1B2	hsa-miR-16-5p
1	ATP8A2	hsa-miR-16-5p
1	ATP9A	hsa-miR-377-3p
1	ATXN7	hsa-miR-92a-3p
1	AXL	hsa-miR-34a-5p
1	B4GALT3	hsa-miR-34a-5p
1	BAG2	hsa-miR-181a-5p
1	BAG3	hsa-miR-221-3p
1	BAG6	hsa-miR-16-5p
1	BAK1	hsa-miR-92a-3p
1	BAMBI	hsa-miR-20a-5p
1	BASP1	hsa-miR-483-3p
1	BAZ2A	hsa-miR-34a-5p
1	BBS10	hsa-miR-32-3p
1	BCAT2	hsa-miR-92a-3p
1	BCL11B	hsa-miR-25-3p
1	BCL2L2	hsa-miR-221-5p
1	BDH2	hsa-miR-766-3p
1	BFAR	hsa-miR-16-5p
1	BIRC5	hsa-miR-16-5p
1	BMP8B	hsa-miR-20a-5p
1	BMPR1A	hsa-miR-92a-3p
1	BNIP3L	hsa-miR-221-3p
1	BRMS1	hsa-miR-221-5p
1	BTF3	hsa-miR-16-5p

1	BTF3L4	hsa-miR-20a-5p
1	BTN3A1	hsa-miR-20a-5p
1	BTN3A3	hsa-miR-20a-5p
1	BYSL	hsa-miR-16-5p
1	C11orf57	hsa-miR-92a-3p
1	C11orf83	hsa-miR-16-5p
1	C16orf70	hsa-miR-20a-5p
1	C1orf115	hsa-miR-877-3p
1	C1orf43	hsa-miR-221-5p
1	C21orf91	hsa-miR-92a-3p
1	C2orf47	hsa-miR-16-5p
1	C3	hsa-miR-766-3p
1	C5orf28	hsa-miR-20a-5p
1	C6orf106	hsa-miR-16-5p
1	C6orf62	hsa-miR-92a-3p
1	C7orf60	hsa-miR-20a-5p
1	C9orf114	hsa-miR-16-5p
1	C9orf78	hsa-miR-20a-5p
1	CAAP1	hsa-miR-16-5p
1	CACNA2D1	hsa-miR-16-5p
1	CAMK2G	hsa-miR-16-5p
1	CAMSAP1	hsa-miR-15a-5p
1	CAPN15	hsa-miR-20a-5p
1	CAPRIN1	hsa-miR-16-5p
1	CASK	hsa-miR-16-5p
1	CASP2	hsa-miR-20a-5p
1	CBFA2T3	hsa-miR-15a-5p
1	CBR1	hsa-miR-93-3p
1	CBX8	hsa-miR-20a-5p
1	CCDC58	hsa-miR-16-5p
1	CCDC59	hsa-miR-16-5p
1	CCNB1	hsa-miR-20a-5p
1	CD69	hsa-miR-92a-3p
1	CDAN1	hsa-miR-34a-5p
1	CDCP1	hsa-miR-665
1	CDIPT	hsa-miR-16-5p
1	CDK12	hsa-miR-766-5p
1	CDK9	hsa-miR-16-5p
1	CDON	hsa-miR-34a-5p
1	CERCAM	hsa-miR-20a-5p
1	CERS2	hsa-miR-20a-5p
1	CFL1	hsa-miR-34a-5p
1	CHD9	hsa-miR-20a-5p
1	CHORDC1	hsa-miR-16-5p
1	CHP1	hsa-miR-16-5p
1	CHPF	hsa-miR-15b-5p
1	CHPT1	hsa-miR-16-5p
1	CHST9	hsa-miR-92a-3p
1	CHSY1	hsa-miR-221-3p
1	CKAP4	hsa-miR-877-3p
1	CLCN3	hsa-miR-15a-5p
1	CLCN6	hsa-miR-15b-5p

1	CLN8	hsa-miR-34a-3p
1	CLOCK	hsa-miR-20a-5p
1	CLTC	hsa-miR-16-5p
1	COG3	hsa-miR-92a-3p
1	COIL	hsa-miR-20a-5p
1	COPS2	hsa-miR-181a-5p
1	CPS1	hsa-miR-20a-5p
1	CPSF6	hsa-miR-221-3p
1	CREB3L2	hsa-miR-92a-3p
1	CRK	hsa-miR-17-5p
1	CROT	hsa-miR-20a-5p
1	CRTC3	hsa-miR-20a-5p
1	CSNK2A1	hsa-miR-337-3p
1	CSTF2T	hsa-miR-221-3p
1	CTSA	hsa-miR-20a-5p
1	CUL2	hsa-miR-574-3p
1	CUX1	hsa-miR-92a-3p
1	CWC22	hsa-miR-877-3p
1	CXorf38	hsa-miR-34b-5p
1	CXXC4	hsa-miR-30b-3p
1	CYB561A3	hsa-miR-16-5p
1	CYB5B	hsa-miR-34a-5p
1	CYBB	hsa-miR-34a-5p
1	CYBRD1	hsa-miR-20a-5p
1	CYP1B1	hsa-miR-221-3p
1	CYP51A1	hsa-miR-15b-5p
1	CYR61	hsa-miR-20a-3p
1	DAB2IP	hsa-miR-92a-3p
1	DBT	hsa-miR-92a-3p
1	DCAF7	hsa-miR-16-5p
1	DCP2	hsa-miR-92a-3p
1	DDAH1	hsa-miR-34a-5p
1	DDX21	hsa-miR-34a-5p
1	DDX52	hsa-miR-16-5p
1	DDX54	hsa-miR-16-5p
1	DENND4B	hsa-miR-92a-3p
1	DENR	hsa-miR-15b-5p
1	DEPDC1	hsa-miR-17-5p
1	DESI1	hsa-miR-16-5p
1	DHCR7	hsa-miR-34a-5p
1	DHFR	hsa-miR-15b-5p
1	DHX30	hsa-miR-16-5p
1	DHX35	hsa-miR-16-5p
1	DHX36	hsa-miR-16-5p
1	DHX8	hsa-miR-16-5p
1	DICER1	hsa-miR-221-3p
1	DKC1	hsa-miR-16-5p
1	DKK2	hsa-miR-221-3p
1	DLK1	hsa-miR-15a-5p
1	DLL1	hsa-miR-34a-5p
1	DMAP1	hsa-miR-16-5p
1	DNAJA2	hsa-miR-16-5p

1	DNAJA4	hsa-miR-16-5p
1	DNAJB12	hsa-miR-92a-3p
1	DNAJB4	hsa-miR-16-5p
1	DNLZ	hsa-miR-197-3p
1	DSG2	hsa-miR-15b-5p
1	DSTYK	hsa-miR-15a-5p
1	DTYMK	hsa-miR-34a-5p
1	E2F4	hsa-miR-377-5p
1	E2F5	hsa-miR-34a-5p
1	EARS2	hsa-miR-92a-3p
1	EDEM3	hsa-miR-34a-5p
1	EEF1A1	hsa-miR-16-5p
1	EFHD2	hsa-miR-34a-5p
1	EFNB1	hsa-miR-34a-5p
1	EFNB2	hsa-miR-16-5p
1	EGFL7	hsa-miR-126-3p
1	EGFR	hsa-miR-16-5p
1	EHBP1	hsa-miR-92a-3p
1	EHD2	hsa-miR-197-3p
1	EIF1AD	hsa-miR-877-3p
1	EIF1AX	hsa-miR-17-3p
1	EIF2AK2	hsa-miR-15b-5p
1	EIF2B2	hsa-miR-16-5p
1	EIF3A	hsa-miR-16-5p
1	EIF3C	hsa-miR-16-5p
1	EIF3CL	hsa-miR-16-5p
1	EIF4A2	hsa-miR-20a-5p
1	EIF4B	hsa-miR-16-5p
1	EIF4EBP1	hsa-miR-93-3p
1	EIF4EBP2	hsa-miR-877-3p
1	EIF4H	hsa-miR-93-3p
1	EIF5A2	hsa-miR-20a-5p
1	ELAVL2	hsa-miR-221-3p
1	ELL2	hsa-miR-15b-3p
1	ELOVL1	hsa-miR-16-5p
1	ELOVL2	hsa-miR-17-3p
1	ELOVL5	hsa-miR-16-5p
1	ELOVL6	hsa-miR-766-3p
1	EMC1	hsa-miR-766-3p
1	EML4	hsa-miR-16-5p
1	EMP1	hsa-miR-34a-5p
1	ENPP4	hsa-miR-16-5p
1	ENTPD6	hsa-miR-16-5p
1	EPB41L4B	hsa-miR-7-1-3p
1	EPHA2	hsa-miR-34a-5p
1	EPT1	hsa-miR-16-5p
1	ERBB2IP	hsa-miR-877-3p
1	ESR1	hsa-miR-221-3p
1	ETS1	hsa-miR-221-3p
1	EVI5L	hsa-miR-34a-5p
1	EXPH5	hsa-miR-877-3p
1	FAF1	hsa-miR-92a-3p

1	FAH	hsa-miR-34a-5p
1	FAM102A	hsa-miR-20a-5p
1	FAM104A	hsa-miR-34a-5p
1	FAM115A	hsa-miR-877-3p
1	FAM117B	hsa-miR-20a-5p
1	FAM122B	hsa-miR-877-3p
1	FAM126B	hsa-miR-92a-3p
1	FAM127A	hsa-miR-766-3p
1	FAM129B	hsa-miR-16-5p
1	FAM155B	hsa-miR-16-5p
1	FAM168A	hsa-miR-16-5p
1	FAM168B	hsa-miR-92a-3p
1	FAM208A	hsa-miR-34a-5p
1	FAM20C	hsa-miR-92a-3p
1	FAM3C	hsa-miR-34a-5p
1	FAM46C	hsa-miR-20a-5p
1	FAM83D	hsa-miR-20a-5p
1	FAM98A	hsa-miR-17-3p
1	FAR1	hsa-miR-93-3p
1	FASTKD2	hsa-miR-16-5p
1	FAT2	hsa-miR-16-5p
1	FBXO33	hsa-miR-181a-5p
1	FBXO41	hsa-miR-16-5p
1	FBXO48	hsa-miR-20a-5p
1	FECH	hsa-miR-16-5p
1	FEN1	hsa-miR-885-5p
1	FGFR1OP	hsa-miR-20a-5p
1	FKBP4	hsa-miR-877-3p
1	FKBP5	hsa-miR-93-3p
1	FMNL3	hsa-miR-20a-5p
1	FOPNL	hsa-miR-92a-3p
1	FOSL1	hsa-miR-34a-5p
1	FOXK2	hsa-miR-20a-5p
1	FOXN3	hsa-miR-34a-5p
1	FOXO3	hsa-miR-197-3p
1	FOXRED2	hsa-miR-20a-5p
1	FRMD6	hsa-miR-20a-5p
1	FUT10	hsa-miR-34a-5p
1	G3BP1	hsa-miR-197-3p
1	GABPA	hsa-miR-92a-3p
1	GALNT3	hsa-miR-221-3p
1	GAPDH	hsa-miR-877-3p
1	GAS1	hsa-miR-34a-5p
1	GCC1	hsa-miR-17-3p
1	GLRX5	hsa-miR-15b-3p
1	GNB1	hsa-miR-16-5p
1	GNPDA1	hsa-miR-34a-5p
1	GNS	hsa-miR-20a-5p
1	GOLGA4	hsa-miR-92a-3p
1	GOLIM4	hsa-miR-877-3p
1	GOLT1B	hsa-miR-16-5p
1	GORASP2	hsa-miR-34a-5p

1	GOT2	hsa-miR-16-5p
1	GPATCH11	hsa-miR-20a-5p
1	GPATCH2L	hsa-miR-629-3p
1	GPATCH4	hsa-miR-16-5p
1	GPR107	hsa-miR-221-3p
1	GPR157	hsa-miR-16-5p
1	GPR180	hsa-miR-16-5p
1	GPR27	hsa-miR-16-5p
1	GPR55	hsa-miR-16-5p
1	GPR83	hsa-miR-181a-5p
1	GPX2	hsa-miR-17-3p
1	GRAMD1B	hsa-miR-92a-3p
1	GREB1	hsa-miR-885-5p
1	GTF2E1	hsa-miR-221-3p
1	GTF2H3	hsa-miR-20a-5p
1	GTF3C2	hsa-miR-16-5p
1	GTF3C3	hsa-miR-16-5p
1	GTF3C4	hsa-miR-16-5p
1	GTPBP8	hsa-miR-16-5p
1	GXYLT1	hsa-miR-92a-3p
1	GYG1	hsa-miR-34a-5p
1	H2AFV	hsa-miR-136-5p
1	H2AFX	hsa-miR-663a
1	H3F3C	hsa-miR-92a-3p
1	HAUS3	hsa-miR-16-5p
1	HDAC1	hsa-miR-34a-5p
1	HDLBP	hsa-miR-34a-5p
1	HEATR1	hsa-miR-16-5p
1	HEATR2	hsa-miR-16-5p
1	HEATR6	hsa-miR-92a-3p
1	HECTD2	hsa-miR-221-3p
1	HEXIM1	hsa-miR-17-3p
1	HEYL	hsa-miR-93-3p
1	HIAT1	hsa-miR-20a-3p
1	HIGD1A	hsa-miR-16-5p
1	HIRIP3	hsa-miR-877-3p
1	HIST1H4B	hsa-miR-34a-5p
1	HIST1H4E	hsa-miR-34a-5p
1	HIST1H4H	hsa-miR-34a-5p
1	HIST2H2BE	hsa-miR-16-5p
1	HIST4H4	hsa-miR-34a-5p
1	HLA-A	hsa-miR-149-3p
1	HMBOX1	hsa-miR-7-1-3p
1	HMGB2	hsa-miR-20a-3p
1	HMGCR	hsa-miR-92a-3p
1	HNRNPA1	hsa-miR-221-5p
1	HNRNPDL	hsa-miR-16-5p
1	HOXA13	hsa-miR-34a-5p
1	HOXB8	hsa-miR-34a-5p
1	HOXC10	hsa-miR-221-3p
1	HOXC4	hsa-miR-30b-3p
1	HOXC8	hsa-miR-92a-3p

1	HSD17B10	hsa-miR-34a-5p
1	HSPA13	hsa-miR-34a-5p
1	HSPA4	hsa-miR-197-3p
1	HSPA4L	hsa-miR-16-5p
1	HSPA5	hsa-miR-16-5p
1	HTT	hsa-miR-16-5p
1	IARS	hsa-miR-16-5p
1	IARS2	hsa-miR-197-3p
1	IER3	hsa-miR-20a-5p
1	IFNAR1	hsa-miR-20a-5p
1	IFNG	hsa-miR-409-3p
1	IFNLR1	hsa-miR-34b-5p
1	IGF2BP1	hsa-miR-877-3p
1	IGF2BP3	hsa-miR-34a-5p
1	IGF2R	hsa-miR-16-5p
1	IKBKG	hsa-miR-15a-5p
1	IKZF2	hsa-miR-92a-3p
1	IKZF5	hsa-miR-20a-5p
1	IL6R	hsa-miR-34a-5p
1	IMPA1	hsa-miR-34a-5p
1	INCENP	hsa-miR-181a-5p
1	INHBB	hsa-miR-34a-5p
1	INPP5F	hsa-miR-17-5p
1	IPMK	hsa-miR-20b-3p
1	IPO7	hsa-miR-16-5p
1	IQGAP1	hsa-miR-136-3p
1	IRS1	hsa-miR-126-3p
1	IRS4	hsa-miR-16-5p
1	ITGA2	hsa-miR-15a-5p
1	ITGA3	hsa-let-7b-3p
1	ITGA5	hsa-miR-92a-3p
1	ITGB8	hsa-miR-20a-5p
1	ITM2B	hsa-miR-25-3p
1	JAG1	hsa-miR-34a-5p
1	JAK1	hsa-miR-20a-5p
1	JAK2	hsa-miR-16-5p
1	JUNB	hsa-miR-663a
1	KAT6A	hsa-miR-877-3p
1	KBTBD6	hsa-miR-34a-5p
1	KBTBD7	hsa-miR-877-3p
1	KCNB1	hsa-miR-106a-5p
1	KCNC4	hsa-miR-16-5p
1	KCNN4	hsa-miR-16-5p
1	KCTD7	hsa-miR-92a-3p
1	KDM2A	hsa-miR-16-5p
1	KDR	hsa-miR-16-5p
1	KHNYN	hsa-miR-877-3p
1	KIAA0226	hsa-miR-16-5p
1	KIAA0368	hsa-miR-16-5p
1	KIAA0754	hsa-miR-877-3p
1	KIAA1432	hsa-miR-16-5p
1	KIAA2013	hsa-miR-16-5p

1	KIF11	hsa-miR-34a-5p
1	KIF14	hsa-miR-16-5p
1	KIF16B	hsa-miR-221-3p
1	KIF1B	hsa-miR-16-5p
1	KIF2A	hsa-miR-16-5p
1	KIF2C	hsa-miR-16-5p
1	KIF3B	hsa-miR-16-5p
1	KIF5A	hsa-miR-16-5p
1	KLF10	hsa-miR-197-3p
1	KLF2	hsa-miR-92a-3p
1	KLHL20	hsa-miR-20a-5p
1	KLHL3	hsa-miR-92a-3p
1	KLHL42	hsa-miR-92a-3p
1	KPNA3	hsa-miR-16-5p
1	KPNA5	hsa-miR-197-3p
1	KRAS	hsa-miR-16-5p
1	KRT10	hsa-miR-20a-5p
1	L1CAM	hsa-miR-16-5p
1	LAMB1	hsa-miR-16-5p
1	LARP4	hsa-miR-16-5p
1	LATS1	hsa-miR-16-5p
1	LATS2	hsa-miR-25-3p
1	LCOR	hsa-miR-92a-3p
1	LDHA	hsa-miR-34a-5p
1	LDOC1	hsa-miR-221-5p
1	LGALS8	hsa-miR-136-5p
1	LIG4	hsa-miR-16-5p
1	LLPH	hsa-miR-34a-5p
1	LMAN1	hsa-miR-34a-3p
1	LMF2	hsa-miR-16-5p
1	LMNB1	hsa-miR-15a-3p
1	LMO7	hsa-miR-16-5p
1	LPHN1	hsa-miR-16-5p
1	LRP6	hsa-miR-221-3p
1	LRRC40	hsa-miR-34a-5p
1	LRRFIP1	hsa-miR-877-3p
1	LURAP1L	hsa-miR-15a-5p
1	LYPLA2	hsa-miR-16-5p
1	LYRM2	hsa-miR-193a-3p
1	LYSMD1	hsa-miR-221-3p
1	MAGEA3	hsa-miR-34a-5p
1	MAGEA6	hsa-miR-34a-5p
1	MAK16	hsa-miR-20a-5p
1	MAN2A1	hsa-miR-92a-3p
1	MAP3K1	hsa-miR-193a-3p
1	MAP3K10	hsa-miR-181a-5p
1	MAP3K5	hsa-miR-20a-5p
1	MAP3K9	hsa-miR-17-5p
1	MAPK6	hsa-miR-19b-1-5p
1	MAPK9	hsa-miR-20a-5p
1	MAPKAPK2	hsa-miR-483-3p
1	MAPRE1	hsa-miR-93-3p

1	MAVS	hsa-miR-20a-5p
1	MBD3	hsa-miR-766-3p
1	MBD6	hsa-miR-34a-5p
1	MBNL2	hsa-miR-369-3p
1	MCM2	hsa-miR-34a-5p
1	MCU	hsa-miR-16-5p
1	MDM4	hsa-miR-34a-5p
1	MDN1	hsa-miR-16-5p
1	MED10	hsa-miR-543
1	MED13	hsa-miR-16-5p
1	MED17	hsa-miR-20a-5p
1	MEGF9	hsa-miR-197-3p
1	MEPCE	hsa-miR-16-5p
1	MESDC2	hsa-miR-15b-5p
1	MET	hsa-miR-34a-5p
1	MFSD8	hsa-miR-20a-5p
1	MIA3	hsa-miR-92a-3p
1	MKRN1	hsa-miR-20a-5p
1	MKRN2	hsa-miR-92a-3p
1	MLXIP	hsa-miR-20a-5p
1	MMP2	hsa-miR-17-5p
1	MORF4L2	hsa-miR-20a-5p
1	MRPS10	hsa-miR-16-5p
1	MRPS2	hsa-miR-16-5p
1	MRPS23	hsa-miR-16-5p
1	MRPS25	hsa-miR-16-5p
1	MSL2	hsa-miR-136-5p
1	MSL3	hsa-miR-16-5p
1	MTA2	hsa-miR-34a-5p
1	MTAP	hsa-miR-34a-5p
1	MTDH	hsa-miR-34a-5p
1	MTMR1	hsa-miR-92a-3p
1	MTMR4	hsa-miR-15b-5p
1	MTMR9	hsa-miR-34a-5p
1	MTOR	hsa-miR-16-5p
1	MTPN	hsa-miR-20a-3p
1	MXI1	hsa-miR-20a-5p
1	MYH14	hsa-miR-877-3p
1	MYO1C	hsa-miR-34a-5p
1	MYO6	hsa-miR-92a-3p
1	NAA30	hsa-miR-15b-5p
1	NAB1	hsa-miR-92a-3p
1	NACC2	hsa-miR-92a-3p
1	NAP1L1	hsa-miR-877-3p
1	NARF	hsa-miR-16-5p
1	NARS	hsa-miR-20a-5p
1	NARS2	hsa-miR-16-5p
1	NCOA1	hsa-miR-34a-5p
1	NCOA3	hsa-miR-20a-5p
1	NEMF	hsa-miR-16-5p
1	NFIC	hsa-miR-16-5p
1	NFKB1	hsa-miR-16-5p

1	NHLRC3	hsa-miR-20a-5p
1	NNT	hsa-miR-16-5p
1	NOA1	hsa-miR-197-3p
1	NOTCH1	hsa-miR-34a-5p
1	NPAT	hsa-miR-20a-5p
1	NPM1	hsa-miR-15b-5p
1	NPNT	hsa-miR-106a-5p
1	NPR3	hsa-miR-16-5p
1	NPTN	hsa-miR-92a-3p
1	NR6A1	hsa-miR-15a-3p
1	NRXN3	hsa-miR-92a-3p
1	NUDT12	hsa-miR-181a-5p
1	NUP155	hsa-miR-92a-3p
1	NUP210	hsa-miR-15b-5p
1	ODC1	hsa-miR-15a-5p
1	OLA1	hsa-miR-877-3p
1	ONECUT2	hsa-miR-16-5p
1	OTUD4	hsa-miR-20a-5p
1	P4HA1	hsa-miR-877-3p
1	PA2G4	hsa-miR-16-5p
1	PABPN1	hsa-miR-629-3p
1	PALM2-AKAP2	hsa-miR-629-3p
1	PANX1	hsa-miR-16-5p
1	PAQR5	hsa-miR-20a-5p
1	PARD6B	hsa-miR-20a-5p
1	PARP4	hsa-miR-877-3p
1	PAX9	hsa-miR-92a-3p
1	PBXIP1	hsa-miR-20a-5p
1	PCGF5	hsa-miR-92a-3p
1	PCMT1	hsa-miR-16-5p
1	PCMTD1	hsa-miR-92a-3p
1	PCNX	hsa-miR-17-5p
1	PDGFRA	hsa-miR-34a-5p
1	PDIK1L	hsa-miR-15b-5p
1	PDPK1	hsa-miR-20a-5p
1	PDRG1	hsa-miR-106a-5p
1	PDZD11	hsa-miR-20a-5p
1	PEA15	hsa-miR-34a-5p
1	PERP	hsa-miR-629-3p
1	PGK1	hsa-miR-20a-5p
1	PHACTR4	hsa-miR-30b-3p
1	PHF12	hsa-miR-30b-3p
1	PHF19	hsa-miR-34a-5p
1	PHF6	hsa-miR-20a-5p
1	PHIP	hsa-miR-16-5p
1	PHLDA2	hsa-miR-193a-3p
1	PIM1	hsa-miR-15b-5p
1	PIP4K2A	hsa-miR-20a-5p
1	PITPNA	hsa-miR-92a-3p
1	PKD2	hsa-miR-17-5p
1	PKP2	hsa-miR-34a-5p
1	PLAGL2	hsa-miR-766-3p

1	PLCG1	hsa-miR-34a-5p
1	PLCL2	hsa-miR-181a-5p
1	PLEC	hsa-miR-16-5p
1	PLEKHO2	hsa-miR-20a-5p
1	PLK2	hsa-miR-126-3p
1	PLRG1	hsa-miR-16-5p
1	PLXNA1	hsa-miR-20a-5p
1	PMAIP1	hsa-miR-197-3p
1	PNP	hsa-miR-16-5p
1	POFUT1	hsa-miR-16-5p
1	POGK	hsa-miR-17-5p
1	POGZ	hsa-miR-34a-5p
1	POLE4	hsa-miR-15a-5p
1	POM121	hsa-miR-877-3p
1	POU3F2	hsa-miR-221-3p
1	PPP1R10	hsa-miR-34a-5p
1	PPP1R3B	hsa-miR-20a-5p
1	PPP2R1A	hsa-miR-16-5p
1	PPP2R1B	hsa-miR-16-5p
1	PPP2R5E	hsa-miR-181a-5p
1	PRKAB2	hsa-miR-16-5p
1	PRKAR1A	hsa-miR-16-5p
1	PRLR	hsa-miR-181a-5p
1	PRPF38B	hsa-miR-877-3p
1	PRPF4	hsa-miR-20a-5p
1	PRR12	hsa-miR-766-5p
1	PRR3	hsa-miR-34a-5p
1	PRRC2C	hsa-miR-16-5p
1	PSAP	hsa-miR-377-3p
1	PSMC2	hsa-miR-16-5p
1	PSMD11	hsa-miR-766-5p
1	PSMD5	hsa-miR-92a-3p
1	PSME4	hsa-miR-16-5p
1	PTBP1	hsa-miR-17-3p
1	PTGER4	hsa-miR-92a-3p
1	PTPLAD1	hsa-miR-20a-3p
1	PTPN3	hsa-miR-16-5p
1	PUM1	hsa-miR-181a-5p
1	PVR	hsa-miR-16-5p
1	PXDN	hsa-miR-377-3p
1	PYGB	hsa-miR-34a-5p
1	QSOX2	hsa-miR-17-3p
1	RAB10	hsa-miR-20a-5p
1	RAB1A	hsa-miR-16-5p
1	RAB2B	hsa-miR-181a-5p
1	RAB8B	hsa-miR-92a-3p
1	RAD21	hsa-miR-92a-3p
1	RAE1	hsa-miR-34a-5p
1	RANGAP1	hsa-miR-16-5p
1	RAP1A	hsa-miR-337-3p
1	RAP2C	hsa-miR-20a-5p
1	RAPGEF6	hsa-miR-766-3p

1	RASEF	hsa-miR-15a-5p
1	RASL10B	hsa-miR-877-3p
1	RASSF2	hsa-miR-16-5p
1	RBBP7	hsa-miR-20a-5p
1	RBCK1	hsa-miR-34a-5p
1	RBL1	hsa-miR-20a-5p
1	RBM12	hsa-miR-34a-5p
1	RBM12B	hsa-miR-20a-5p
1	RBMS1	hsa-miR-16-5p
1	RBMS3	hsa-miR-16-5p
1	RCCD1	hsa-miR-20a-5p
1	RCL1	hsa-miR-16-5p
1	RDH11	hsa-miR-34a-5p
1	REEP5	hsa-miR-20a-5p
1	RET	hsa-miR-15a-5p
1	REXO1	hsa-miR-92a-3p
1	RFC3	hsa-miR-20a-5p
1	RFFL	hsa-miR-92a-3p
1	RFT1	hsa-miR-16-5p
1	RFX1	hsa-miR-197-3p
1	RGP1	hsa-miR-34a-5p
1	RGS3	hsa-miR-126-3p
1	RIMS3	hsa-miR-16-5p
1	RLF	hsa-miR-181a-5p
1	RNF11	hsa-miR-17-3p
1	RNF111	hsa-miR-16-5p
1	RNF144B	hsa-miR-16-5p
1	RNF219	hsa-miR-16-2-3p
1	RNF34	hsa-miR-20a-5p
1	RNF40	hsa-miR-34a-5p
1	RP2	hsa-miR-92a-3p
1	RPA2	hsa-miR-20a-5p
1	RPIA	hsa-miR-34a-5p
1	RPL13	hsa-miR-16-5p
1	RPL13A	hsa-miR-877-3p
1	RPL28	hsa-miR-766-3p
1	RPL37A	hsa-miR-15a-3p
1	RPL5	hsa-miR-16-5p
1	RPL7A	hsa-miR-197-3p
1	RPRD1B	hsa-miR-16-5p
1	RPS16	hsa-let-7b-3p
1	RPS17	hsa-miR-16-5p
1	RPS24	hsa-miR-92a-3p
1	RPS27	hsa-miR-16-5p
1	RRAS	hsa-miR-34a-5p
1	RRAS2	hsa-miR-20a-5p
1	RRM2	hsa-miR-20a-5p
1	RSRC2	hsa-miR-197-3p
1	S1PR3	hsa-miR-766-3p
1	SART1	hsa-miR-16-5p
1	SBNO2	hsa-miR-25-3p
1	SCAF4	hsa-miR-16-5p

1	SCAMP3	hsa-miR-16-5p
1	SCCPDH	hsa-miR-15b-5p
1	SCD	hsa-miR-20a-5p
1	SDC2	hsa-miR-7-1-3p
1	SDE2	hsa-miR-34a-3p
1	SDHAF2	hsa-miR-16-5p
1	SEC23A	hsa-miR-20a-5p
1	SEC24A	hsa-miR-16-5p
1	SEC61A1	hsa-miR-16-5p
1	SEC61A2	hsa-miR-16-5p
1	SECISBP2L	hsa-miR-647
1	SERBP1	hsa-miR-16-5p
1	SERINC1	hsa-miR-106a-5p
1	SESN1	hsa-miR-377-3p
1	SF1	hsa-miR-17-3p
1	SF3A3	hsa-miR-16-5p
1	SFXN1	hsa-miR-16-5p
1	SGPL1	hsa-miR-20a-5p
1	SH3PXD2A	hsa-miR-92a-3p
1	SIK2	hsa-miR-181a-5p
1	SIKE1	hsa-miR-20a-5p
1	SIPA1L2	hsa-miR-16-5p
1	SIRT1	hsa-miR-34a-5p
1	SIX4	hsa-miR-885-5p
1	SLAIN2	hsa-miR-20a-5p
1	SLC12A2	hsa-miR-16-5p
1	SLC12A7	hsa-miR-766-3p
1	SLC16A2	hsa-miR-17-5p
1	SLC16A3	hsa-miR-16-5p
1	SLC1A1	hsa-miR-629-3p
1	SLC20A1	hsa-miR-379-5p
1	SLC25A28	hsa-miR-20a-5p
1	SLC25A37	hsa-miR-17-5p
1	SLC30A7	hsa-miR-106a-5p
1	SLC35A4	hsa-miR-16-5p
1	SLC35G2	hsa-miR-34a-5p
1	SLC38A7	hsa-miR-30b-3p
1	SLC39A14	hsa-miR-16-5p
1	SLC7A1	hsa-miR-16-5p
1	SLITRK1	hsa-miR-24-1-5p
1	SLK	hsa-miR-20a-5p
1	SMAD3	hsa-miR-16-5p
1	SMARCA5	hsa-miR-25-3p
1	SMARCC1	hsa-miR-34a-5p
1	SMC1A	hsa-miR-93-3p
1	SMEK2	hsa-miR-16-5p
1	SMIM13	hsa-miR-20a-5p
1	SMS	hsa-miR-7-1-3p
1	SMU1	hsa-miR-766-3p
1	SNAI1	hsa-miR-34a-5p
1	SNAI2	hsa-miR-181a-5p
1	SNN	hsa-miR-92a-3p

1	SNRNP200	hsa-miR-877-3p
1	SNRPB2	hsa-miR-16-5p
1	SNRPC	hsa-miR-16-5p
1	SNX12	hsa-miR-16-5p
1	SOCS4	hsa-miR-34a-5p
1	SOCS5	hsa-miR-20a-5p
1	SOD1	hsa-miR-377-3p
1	SON	hsa-miR-16-5p
1	SOWAHC	hsa-miR-16-5p
1	SOX11	hsa-miR-197-3p
1	SOX2	hsa-miR-126-3p
1	SP1	hsa-miR-16-5p
1	SPATA2	hsa-miR-16-5p
1	SPOPL	hsa-miR-20a-5p
1	SPRYD3	hsa-miR-16-5p
1	SPTAN1	hsa-miR-93-3p
1	SPTSSA	hsa-miR-221-3p
1	SRC	hsa-miR-34a-5p
1	SREK1IP1	hsa-miR-92a-3p
1	SRGN	hsa-miR-181a-5p
1	SRP72	hsa-miR-16-5p
1	SRPK1	hsa-miR-16-5p
1	SRSF1	hsa-miR-377-3p
1	SS18	hsa-miR-197-3p
1	SSH2	hsa-miR-20a-5p
1	SSR1	hsa-miR-30b-3p
1	STAMBP	hsa-miR-221-3p
1	STARD7	hsa-miR-193a-3p
1	STEAP3	hsa-miR-16-5p
1	STIM1	hsa-miR-17-3p
1	STK17B	hsa-miR-20a-5p
1	STMN1	hsa-miR-221-3p
1	STX17	hsa-miR-15a-5p
1	STX7	hsa-miR-543
1	SUB1	hsa-miR-16-2-3p
1	SUCLA2	hsa-miR-16-5p
1	SUCO	hsa-miR-20a-5p
1	SUMO1	hsa-miR-877-3p
1	SURF4	hsa-miR-34a-5p
1	SV2A	hsa-miR-92a-3p
1	SYF2	hsa-miR-16-5p
1	SYNGR2	hsa-miR-34a-5p
1	SYT1	hsa-miR-34a-5p
1	TAF15	hsa-miR-877-3p
1	TANC1	hsa-miR-20a-5p
1	TANC2	hsa-miR-92a-3p
1	TANGO6	hsa-miR-15b-5p
1	TARDBP	hsa-miR-93-3p
1	TBC1D1	hsa-miR-7-1-3p
1	TBC1D15	hsa-miR-17-5p
1	TBL3	hsa-miR-16-5p
1	TCF4	hsa-miR-20a-5p

1	TCFL5	hsa-miR-16-5p
1	TECPR2	hsa-miR-92a-3p
1	TELO2	hsa-miR-16-5p
1	TES	hsa-let-7b-3p
1	TFAM	hsa-miR-20a-5p
1	TFAP4	hsa-miR-16-5p
1	TGFBI	hsa-miR-16-5p
1	TGFBR2	hsa-miR-20a-5p
1	THRA	hsa-miR-15b-5p
1	TIAM1	hsa-miR-221-3p
1	TIMM10B	hsa-miR-16-5p
1	TIMP2	hsa-miR-106a-5p
1	TIMP3	hsa-miR-17-5p
1	TIPARP	hsa-miR-221-3p
1	TJP1	hsa-miR-379-3p
1	TMBIM6	hsa-miR-20a-5p
1	TMED4	hsa-miR-181a-5p
1	TMED7	hsa-miR-221-3p
1	TMEM154	hsa-miR-16-5p
1	TMEM248	hsa-miR-221-3p
1	TMEM30A	hsa-miR-181a-5p
1	TMEM41A	hsa-miR-16-5p
1	TMEM87A	hsa-miR-16-5p
1	TMOD2	hsa-miR-34a-5p
1	TMTC1	hsa-miR-665
1	TMTC3	hsa-miR-16-5p
1	TMX3	hsa-miR-20a-5p
1	TMX4	hsa-miR-197-3p
1	TNFAIP1	hsa-miR-20a-5p
1	TNFRSF1B	hsa-miR-193a-3p
1	TNFRSF21	hsa-miR-20a-5p
1	TNPO3	hsa-miR-16-5p
1	TNRC18	hsa-miR-34a-5p
1	TOMM20	hsa-miR-93-3p
1	TOMM34	hsa-miR-16-5p
1	TOPORS	hsa-miR-20a-5p
1	TOR4A	hsa-miR-16-5p
1	TP53	hsa-miR-34a-5p
1	TPD52	hsa-miR-34a-5p
1	TPGS1	hsa-miR-766-3p
1	TPM3	hsa-miR-16-5p
1	TPPP	hsa-miR-34a-5p
1	TPPP3	hsa-miR-16-5p
1	TRABD	hsa-miR-17-3p
1	TRAF4	hsa-miR-16-5p
1	TRAPPC2	hsa-miR-92a-3p
1	TRIB1	hsa-miR-877-3p
1	TRIM2	hsa-miR-493-5p
1	TRIM25	hsa-miR-877-3p
1	TRIM44	hsa-miR-16-5p
1	TRPS1	hsa-miR-221-3p
1	TRUB1	hsa-miR-15a-3p

1	TSPAN13	hsa-miR-221-3p
1	TSPYL1	hsa-miR-877-3p
1	TSR1	hsa-miR-16-5p
1	TTC17	hsa-miR-16-5p
1	TTC33	hsa-let-7b-3p
1	TTL	hsa-miR-877-3p
1	TTLL12	hsa-miR-16-5p
1	TTPAL	hsa-miR-20a-5p
1	TTYH3	hsa-miR-7-1-3p
1	TUB	hsa-miR-221-3p
1	TUBGCP2	hsa-miR-16-5p
1	TUBGCP3	hsa-miR-483-3p
1	TXN2	hsa-miR-16-5p
1	TXNIP	hsa-miR-34a-5p
1	TXNRD2	hsa-miR-17-3p
1	TYMS	hsa-miR-34a-5p
1	UBAP2	hsa-miR-34a-5p
1	UBC	hsa-miR-20a-5p
1	UBE2G1	hsa-miR-17-3p
1	UBE2H	hsa-miR-17-3p
1	UBE2V1	hsa-miR-16-5p
1	UBE4A	hsa-miR-16-5p
1	UBP1	hsa-miR-34a-5p
1	UBXN2A	hsa-miR-20a-5p
1	UBXN2B	hsa-miR-34a-5p
1	UBXN7	hsa-miR-877-3p
1	UEVLD	hsa-miR-20a-5p
1	UFC1	hsa-miR-16-5p
1	UGT8	hsa-miR-16-5p
1	ULBP2	hsa-miR-34a-5p
1	UNG	hsa-miR-16-5p
1	UNK	hsa-miR-17-3p
1	UPF2	hsa-miR-92a-3p
1	USP28	hsa-miR-221-3p
1	USP47	hsa-miR-34a-5p
1	UTP15	hsa-miR-16-5p
1	UTP20	hsa-miR-16-5p
1	VAMP2	hsa-miR-34a-5p
1	VAMP8	hsa-miR-16-5p
1	VASN	hsa-miR-34a-5p
1	VBP1	hsa-miR-92a-3p
1	VCAM1	hsa-miR-126-3p
1	VCL	hsa-miR-34a-5p
1	VCPIP1	hsa-miR-92a-3p
1	VEZF1	hsa-miR-20a-5p
1	VIM	hsa-miR-17-3p
1	VLDLR	hsa-miR-409-5p
1	VMA21	hsa-miR-16-5p
1	VMP1	hsa-miR-16-5p
1	VPS13A	hsa-miR-15b-5p
1	VPS33B	hsa-miR-16-5p
1	VPS37B	hsa-miR-34a-5p

1	VTI1B	hsa-miR-16-5p
1	WAC	hsa-miR-20a-5p
1	WBP11	hsa-miR-16-5p
1	WDR26	hsa-miR-92a-3p
1	WDR43	hsa-miR-16-5p
1	XKR8	hsa-miR-16-5p
1	XPNPEP3	hsa-miR-16-5p
1	XPO6	hsa-miR-16-5p
1	XPO7	hsa-miR-16-5p
1	XPOT	hsa-miR-16-5p
1	YARS	hsa-miR-877-3p
1	YARS2	hsa-miR-16-5p
1	YBX1	hsa-miR-34a-5p
1	YIPF4	hsa-miR-16-5p
1	YIPF5	hsa-miR-92a-3p
1	YWHAE	hsa-miR-32-3p
1	YY1	hsa-miR-34a-5p
1	ZBTB2	hsa-miR-16-5p
1	ZBTB47	hsa-miR-106a-5p
1	ZBTB6	hsa-miR-20a-5p
1	ZBTB9	hsa-miR-20a-5p
1	ZC3H4	hsa-miR-34a-5p
1	ZC3HAV1	hsa-miR-92a-3p
1	ZCCHC24	hsa-miR-30b-3p
1	ZDHHC16	hsa-miR-34a-5p
1	ZDHHC21	hsa-miR-25-3p
1	ZDHHC7	hsa-miR-877-3p
1	ZKSCAN8	hsa-miR-221-3p
1	ZMIZ1	hsa-miR-493-5p
1	ZNF100	hsa-miR-766-3p
1	ZNF12	hsa-miR-181a-5p
1	ZNF134	hsa-miR-25-3p
1	ZNF207	hsa-miR-16-5p
1	ZNF217	hsa-miR-15b-5p
1	ZNF275	hsa-miR-221-3p
1	ZNF280C	hsa-miR-16-5p
1	ZNF281	hsa-miR-136-5p
1	ZNF292	hsa-miR-32-3p
1	ZNF302	hsa-miR-197-3p
1	ZNF354B	hsa-miR-92a-3p
1	ZNF430	hsa-miR-92a-3p
1	ZNF431	hsa-miR-16-2-3p
1	ZNF440	hsa-miR-181a-5p
1	ZNF503	hsa-miR-92a-3p
1	ZNF507	hsa-miR-92a-3p
1	ZNF594	hsa-miR-181a-5p
1	ZNF598	hsa-miR-92a-3p
1	ZNF623	hsa-miR-34a-5p
1	ZNF629	hsa-miR-877-3p
1	ZNF652	hsa-miR-221-3p
1	ZNF706	hsa-miR-20a-5p
1	ZNF721	hsa-miR-92a-3p

1	ZNF732	hsa-miR-766-3p
1	ZNF785	hsa-miR-92a-3p
1	ZNF792	hsa-miR-7-1-3p
1	ZNF800	hsa-miR-20a-5p
1	ZNF827	hsa-miR-16-5p
1	ZRANB1	hsa-miR-20a-5p
1	ZYG11B	hsa-miR-197-3p
1	ZYX	hsa-miR-16-5p
