# Supplementary material for: Deregulation of the imprinted DLK1-DIO3 locus ncRNAs is associated with replicative senescence of human adipose-derived stem cells
Source: PLoS One. 2018 Nov 5;13(11):e0206534. doi: 10.1371/journal.pone.0206534 (PMC6218046; doi:10.1371/journal.pone.0206534)
Supplement: S3 Table — (DOCX) [file pone.0206534.s007.docx]

| **GO ID** | **GO Term** | **P-Value** | **Fold Enrichment** | **FDR** |
| --- | --- | --- | --- | --- |
|  |  |  |  |  |
| GO:0045944 | Positive regulation of transcription from RNA polymerase II promoter | 1,82E-12 | 1,81 | 3,44013E-09 |
| GO:0045893 | Positive regulation of transcription, DNA-templated | 2,00E-10 | 2,06 | 3,77613E-07 |
| GO:0016032 | Viral process | 1,04E-09 | 2,32 | 1,96923E-06 |
| GO:0098609 | Cell-cell adhesion | 2,54E-09 | 2,44 | 4,78904E-06 |
| GO:0043066 | Negative regulation of apoptotic process | 1,70E-08 | 1,99 | 3,19537E-05 |
| GO:1900034 | Regulation of cellular response to heat | 4,91E-08 | 3,84 | 9,25371E-05 |
| GO:0006366 | Transcription from RNA polymerase II promoter | 2,72E-07 | 1,84 | 0,001 |
| GO:0042493 | Response to drug | 1,07E-06 | 1,99 | 0,002 |
| GO:0042771 | Intrinsic apoptotic signaling pathway in response to DNA damage by p53 | 1,41E-06 | 5,43 | 0,003 |
| GO:0008283 | Cell proliferation | 1,86E-06 | 1,95 | 0,003 |
| GO:0045892 | Negative regulation of transcription, DNA-templated | 2,34E-06 | 1,78 | 0,004 |
| GO:0010629 | Negative regulation of gene expression | 4,14E-06 | 2,61 | 0,008 |
| GO:0006977 | DNA damage response in cell cycle arrest | 4,52E-06 | 3,64 | 0,009 |
| GO:0006351 | Transcription, DNA-templated | 6,77E-06 | 1,33 | 0,013 |
| GO:0043161 | Proteasome-mediated ubiquitin-dependent protein catabolic process | 9,01E-06 | 2,24 | 0,017 |
| GO:0050821 | Protein stabilization | 2,08E-05 | 2,51 | 0,039 |
| GO:0008285 | Negative regulation of cell proliferation | 2,43E-05 | 1,78 | 0,046 |
| GO:0007265 | Ras protein signal transduction | 2,61E-05 | 3,22 | 0,049 |
| GO:0006413 | Translational initiation | 3,53E-05 | 2,43 | 0,067 |
| GO:0016239 | Positive regulation of macroautophagy | 3,72E-05 | 5,45 | 0,070 |
| GO:0006457 | Protein folding | 3,99E-05 | 2,21 | 0,075 |
| GO:0000082 | G1/S transition of mitotic cell cycle | 4,56E-05 | 2,70 | 0,086 |
| GO:0006468 | Protein phosphorylation | 4,68E-05 | 1,70 | 0,088 |
| GO:0008284 | Positive regulation of cell proliferation | 6,16E-05 | 1,67 | 0,116 |
| GO:0030512 | Negative regulation of TGFRβ signaling pathway | 8,06E-05 | 3,23 | 0,152 |
| GO:0071456 | Cellular response to hypoxia | 9,48E-05 | 2,58 | 0,179 |
| GO:0009612 | Response to mechanical stimulus | 1,19E-04 | 3,13 | 0,224 |
| GO:0070059 | Intrinsic apoptotic signaling pathway in response to ER stress | 1,27E-04 | 4,31 | 0,240 |
| GO:0045429 | Positive regulation of nitric oxide biosynthetic process | 1,52E-04 | 3,62 | 0,287 |
| GO:0000122 | Negative regulation of transcription from RNA polymerase II promoter | 1,68E-04 | 1,49 | 0,316 |
| GO:0097192 | Extrinsic apoptotic signaling pathway in absence of ligand | 1,70E-04 | 4,18 | 0,320 |
| GO:0007050 | Cell cycle arrest | 1,73E-04 | 2,26 | 0,325 |
| GO:0016575 | Histone deacetylation | 1,91E-04 | 3,54 | 0,360 |
| GO:0006915 | Apoptotic process | 2,81E-04 | 1,54 | 0,528 |
| GO:0000186 | Activation of MAPKK activity | 2,95E-04 | 3,39 | 0,554 |
| GO:0051726 | Regulation of cell cycle | 3,02E-04 | 2,32 | 0,567 |
| GO:0040008 | Regulation of growth | 3,04E-04 | 3,19 | 0,572 |
| GO:0046777 | Protein autophosphorylation | 3,33E-04 | 2,06 | 0,626 |
| GO:0070301 | Cellular response to hydrogen peroxide | 4,27E-04 | 2,94 | 0,801 |
| GO:0006892 | Post-Golgi vesicle-mediated transport | 4,33E-04 | 5,27 | 0,812 |
| GO:0032570 | Response to progesterone | 4,43E-04 | 3,26 | 0,832 |
| GO:0030335 | Positive regulation of cell migration | 4,46E-04 | 2,00 | 0,838 |
| GO:0006928 | Movement of cell or subcellular component | 4,50E-04 | 2,59 | 0,844 |
| GO:0018107 | Peptidyl-threonine phosphorylation | 4,76E-04 | 3,72 | 0,894 |
| GO:0075733 | Intracellular transport of virus | 5,38E-04 | 3,19 | 1,009 |
| GO:0046902 | Regulation of mitochondrial membrane permeability | 5,74E-04 | 7,52 | 1,077 |
| GO:0051146 | striated muscle cell differentiation | 5,74E-04 | 7,52 | 1,077 |
| GO:0006461 | Protein complex assembly | 8,11E-04 | 2,27 | 1,518 |
| GO:0016925 | Protein sumoylation | 8,11E-04 | 2,27 | 1,518 |
| GO:0097150 | Neuronal stem cell population maintenance | 8,66E-04 | 4,77 | 1,621 |
| GO:0007569 | Cell aging | 9,01E-04 | 4,18 | 1,684 |
| GO:0045930 | Negative regulation of mitotic cell cycle | 9,01E-04 | 4,18 | 1,684 |
| GO:0001934 | Positive regulation of protein phosphorylation | 9,03E-04 | 2,15 | 1,689 |
| GO:0071260 | Cellular response to mechanical stimulus | 0,001 | 2,51 | 1,869 |
| GO:0042787 | Protein ubiquitination involved in ubiquitin-dependent protein catabolic process | 0,001 | 2,05 | 1,962 |
| GO:0018108 | Peptidyl-tyrosine phosphorylation | 0,001 | 2,03 | 2,151 |
| GO:0000209 | Protein polyubiquitination | 0,001 | 1,92 | 2,549 |
| GO:2000811 | Negative regulation of anoikis | 0,001 | 5,16 | 2,719 |
| GO:0048008 | Platelet-derived growth factor receptor signaling pathway | 0,002 | 3,89 | 2,806 |
| GO:0006661 | Phosphatidylinositol biosynthetic process | 0,002 | 2,81 | 3,336 |
| GO:0007049 | Cell cycle | 0,002 | 1,80 | 3,749 |
| GO:0006446 | Regulation of translational initiation | 0,002 | 3,39 | 3,783 |
| GO:0045737 | Positive regulation of cyclin-dependent protein serine/threonine kinase activity | 0,002 | 4,18 | 3,861 |
| GO:0022604 | Regulation of cell morphogenesis | 0,003 | 4,01 | 4,971 |
| GO:0034644 | Cellular response to UV | 0,003 | 3,00 | 5,309 |
| GO:0006409 | tRNA export from nucleus | 0,003 | 3,52 | 5,479 |
| GO:0000462 | Maturation of SSU-rRNA from tricistronic rRNA transcript | 0,003 | 3,52 | 5,479 |
| GO:0030522 | Intracellular receptor signaling pathway | 0,003 | 3,21 | 5,531 |
| GO:0043154 | Negative regulation of cysteine-type endopeptidases in apoptotic process | 0,003 | 2,51 | 5,968 |
| GO:0008630 | Intrinsic apoptotic signaling pathway in response to DNA damage | 0,003 | 2,93 | 6,242 |
| GO:0042981 | Regulation of apoptotic process | 0,003 | 1,76 | 6,273 |
| GO:0043065 | Positive regulation of apoptotic process | 0,004 | 1,60 | 6,585 |
| GO:0045727 | Positive regulation of translation | 0,004 | 2,73 | 6,636 |
| GO:0010863 | Positive regulation of phospholipase C activity | 0,004 | 6,96 | 6,684 |
| GO:0034333 | Adherens junction assembly | 0,004 | 6,96 | 6,684 |
| GO:0072583 | clathrin-mediated endocytosis | 0,004 | 4,38 | 6,784 |
| GO:0032436 | Positive regulation of proteasomal ubiquitin-dependent catabolic process | 0,004 | 2,59 | 6,786 |
| GO:0043433 | Negative regulation of sequence-specific DNA binding transcription factors | 0,004 | 2,59 | 6,786 |
| GO:0019083 | Viral transcription | 0,004 | 2,09 | 7,136 |
| GO:0001701 | In utero embryonic development | 0,004 | 1,80 | 7,475 |
| GO:0006606 | Protein import into nucleus | 0,004 | 2,68 | 7,637 |
| GO:0007173 | Epidermal growth factor receptor signaling pathway | 0,004 | 2,68 | 7,637 |
| GO:0071407 | Cellular response to organic cyclic compound | 0,004 | 2,54 | 7,723 |
| GO:0007219 | Notch signaling pathway | 0,004 | 2,07 | 7,819 |
| GO:0014031 | Mesenchymal cell development | 0,004 | 10,02 | 8,114 |
| GO:0048015 | Phosphatidylinositol-mediated signaling | 0,005 | 2,11 | 8,435 |
| GO:0045766 | Positive regulation of angiogenesis | 0,005 | 2,05 | 8,552 |
| GO:1900087 | Positive regulation of G1/S transition of mitotic cell cycle | 0,005 | 4,18 | 8,776 |
| GO:0031397 | Negative regulation of protein ubiquitination | 0,005 | 2,98 | 9,207 |
| GO:0007030 | Golgi organization | 0,005 | 2,37 | 9,609 |
| GO:0097421 | Liver regeneration | 0,005 | 3,22 | 9,694 |
