# Supplementary material for: Deregulation of the imprinted DLK1-DIO3 locus ncRNAs is associated with replicative senescence of human adipose-derived stem cells
Source: PLoS One. 2018 Nov 5;13(11):e0206534. doi: 10.1371/journal.pone.0206534 (PMC6218046; doi:10.1371/journal.pone.0206534)
Supplement: S1 File — Extended methods. (DOCX) [file pone.0206534.s009.docx]

**SUPPLEMENTARY METHODS**

**hADSC cell origin and isolation.** aADSC cultures were acquired from Inbiobank Stem Cell Bank ([www.inbiobank.org](http://www.inbiobank.org)) or were established from lipoaspirates, kindly provided by Dr. Damian García-Olmo (Hospital Universitario La Paz, Madrid, Spain). pADSC cultures were derived from pediatric surgery resections, kindly provided by Dr. Manuel Ramirez Orellana (Hospital Infantil Universitario Niño Jesús, Madrid, Spain). Briefly, samples from lipoaspirates (aADSC) or pediatric surgical resection (pADSC) were mechanically dissociated, followed by digestion with collagenase P (1 mg/ml; Roche Diagnostics) in Dulbecco’s modified Eagle’s medium (DMEM; Sigma-Aldrich) for 2 h at 37ºC, with gentle rocking. Collagenase was then neutralized with fetal bovine serum (FBS; Sigma; 10% final concentration) and ADSCs were sedimented, resuspended in complete DMEM supplemented with 10% FBS, 2 mM glutamine, penicillin (100 U/ml)/streptomycin (1000 U/ml), and clarified through a 40- µm nylon filter (Becton-Dickinson) before seeding [1].

**hADSC culture.** hADSCs were cultured as described [2,3]. Briefly, cells were cultured at a density of 2 × 10^3^ cells/cm^2^ in DMEM supplemented with 10% FBS, 2 mM glutamine, penicillin (100 U/ml)/streptomycin (1000 U/ml) (37ºC, 5% CO_2_). Long-term cell growth was monitored by manual cell counting with a hemocytometer. Cumulative population doubling (PD) was calculated with the formula: PD = (log (fN/iN))/log 2, where fN is the final cell number and iN is initial cell number. We defined prolonged culture conditions as (P_L_), (≥20 passages; but variable with the individual isolates) and short culture conditions (P_S_) (≤12 passages).

**hADSC surface marker characterization.** Cell surface markers were analyzed as previously described [2,4,5] by flow-cytometry. Briefly, 100,000 cells where incubated for 15 min at room temperature in 1×PBS supplemented with 0.1 BSA and 1% human serum. Samples were stained with the following antibodies: CD31-ALEXA488 (BD Bioscience), CD34-PE (DAKO) CD29-PECy5 (BD Bioscience), CD44-APC (BD Bioscience), CD45-PerCp (BD Bioscience), CD166-PE (BD Bioscience), CD177-APC (DAKO), and CD47 (Santa Cruz Biotechnology, Inc.), conjugated with ALEXA-647 (Molecular Probes). Data acquisition and analysis was performed with FacsDiva Software (BD Bioscience).

**hADSC cell differentiation assay.** hADSCs were seeded at 5 × 10^3^ cells/cm^2^ in complete DMEM. Once the culture reached ~80% confluence (24–48 h after seeding), the medium was replaced with specific induction medium. For adipose lineage induction, aMEM was supplemented with 10% FBS, 1 µM dexamethasone, 0.5 mM IBMX (3-isobutyl-1-methyl xanthine), 10 µg/mL insulin and 100 µM indomethacin. Adipose-differentiated cultures were fixed with 4% paraformaldehyde and stained with Oil Red O (Amresco). For osteogenic lineage induction, αMEM was supplemented with 10% FBS, 0.1 µM dexamethasone, 0.2 mM ascorbic acid 2-phosphate, and 10 mM glycerol 2-phosphate. Osteogenic-differentiated cultures were fixed with 70% ethanol and stained with Alizarin Red S (Sigma-Aldrich).

**mADSC cell origin.** Mouse ADSC cultures were kindly provided by Drs. Maruja Lamana and Rosa Yañez (División de Terapias Innovadoras, CIEMAT, Madrid, Spain). Cultures were established and passaged as described [6].

**Other cell models.** Human foreskin fibroblasts, embryonic stem cell lines, non-immortalized forebrain human neural progenitor cells (hNPC) and human v-myc-immortalized NPCs were cultured as described [7,8].

**Cell cycle analysis.** For cell cycle distribution analysis, long- (P_L_) and short-term (P_S_) aADSC cultures were stained with propidium iodide (PI). Briefly, 2 × 10^5^ cells were fixed with 70% ethanol, washed with PBS, treated with 100 µg/ml RNase A (Sigma-Aldrich; 1 h, 37ºC), and stained with 0.005% PI in PBS. Cells were analyzed on a FACS-Canto II cytometer (Becton-Dickinson); fluorescence intensity data was acquired with FacsDIVA software (BD Bioscience) and downstream analysis was performed with ModFit software (Verity Software House).

**Cell senescence-associated ß-galactosidase staining.** hADSCs were seeded at 5 × 10^3^ cells/cm^2^ in complete DMEM. Once the culture reached ~80% confluence, samples where stained for SA-ß-gal using the Cellular Senescence Assay Kit (Chemicon International). Briefly, cells were washed with 1×PBS and fixed at room temperature. Cells were then incubated with SA-ß-gal staining solution (provided by the kit), at 37^o^C, protected from light. Stained samples were washed with 1×PBS and positive staining was evaluated by light microscopy.

**microRNA RT-qPCR analysis.** TaqMan MicroRNA Assays (Applied Biosystems) were used for expression analysis of hsa-miR-34a, hsa-miR-136 and hsa-miR-369-5p, with U6 snRNA for normalization. A list of probes is shown in Supplementary Table S3.

**Epigenetic drug treatment of hADSCs.** Epigenetic drugs used in were 5 µM 5-aza-2’-deoxycytidine (AZA; Calbiochem) or 75 nM trichostatin A (TSA; Sigma). hADSCs were exposed to drugs for 72 h and the cultures were maintained for an additional 96 h without drugs.

**Bioinformatic analysis of miRNA-validated targets.** For bioinformatics analyses, only mature forms of miRNA probes were used. Validated targets for the different miRNAs were downloaded from TarBase v7.0 (TarBase Score >0) [9], miRTarBase [10] and miRWalk 2.0 [11]. Only miRNA-target interactions identified by all three databases were considered for downstream analysis.

**References**

1. Garcia-Olmo, D et al. 2005. A phase I clinical trial of the treatment of Crohn's fistula by adipose mesenchymal stem cell transplantation. Dis Colon Rectum 48:1416-23.

2. Estrada JC, Albo C, Benguría A, Dopazo A, López-Romero P, Carrera-Quintanar L, et al. Culture of human mesenchymal stem cells at low oxygen tension improves growth and genetic stability by activating glycolysis. Cell Death Differ. 2012; 9:743-755.

3. Fathi E, Farahzadi R (2017) Enhancement of osteogenic differentiation of rat adipose tissue-derived mesenchymal stem cells by zinc sulphate under electromagnetic field via the PKA, ERK1/2 and  Wnt/-catenin signaling pathways. PLoS ONE 12(3): e0173877.

4. Sepúlveda JC, Tomé M, Fernández ME, Delgado M, Campisi J, Bernad A, et al. [Cell senescence abrogates the therapeutic potential of human mesenchymal stem cells in the lethal endotoxemia model.](https://www.ncbi.nlm.nih.gov/pubmed/24496748) Stem Cells 2014; 32:1865-77

5.Farahzadi R, Fathi E, Mesbah-Namin SA, Zarghami N (2017) Zinc sulfate contributes to promote telomere length extension via increasing telomerase gene expression, telomerase activity and change in the TERT gene promoter CpG island methylation status of human adipose-derived mesenchymal stem cells. PLoS ONE 12(11):e0188052. doi: 10.1371.

6. Yanez, R et al. 2006. Adipose tissue-derived mesenchymal stem cells have in vivo immunosuppressive properties applicable for the control of the graft-versus-host disease. Stem Cells 24: 2582-91

7. Pino-Barrio, MJ et al. 2015. V-myc immortalizes human neural stem cells in the absence of pluripotency-associated traits. PLoS One 10: e0118499.

8. Villa, A et al. 2004. Long-term molecular and cellular stability of human neural stem cell lines. Exp Cell Res 294: 559-70.

9.Vlachos, IS et al. 2015. DIANA-TarBase v7.0: indexing more than half a million experimentally supported miRNA:mRNA interactions. Nucleic Acids Res 43:D153-9.

10. Chou, CH et al. 2016. miRTarBase 2016: updates to the experimentally validated miRNA-target interactions database. Nucleic Acids Res 44:D239-47.

11. Dweep, H and Gretz, N, 2015. miRWalk2.0: a comprehensive atlas of microRNA-target interactions. Nat Methods 12: 697.

.
